# The ecology, subsistence and diet of ~45,000-year-old *Homo sapiens* at Ilsenhöhle in Ranis, Germany

**DOI:** 10.1038/s41559-023-02303-6

**Published:** 2024-01-31

**Authors:** Geoff M. Smith, Karen Ruebens, Elena Irene Zavala, Virginie Sinet-Mathiot, Helen Fewlass, Sarah Pederzani, Klervia Jaouen, Dorothea Mylopotamitaki, Kate Britton, Hélène Rougier, Mareike Stahlschmidt, Matthias Meyer, Harald Meller, Holger Dietl, Jörg Orschiedt, Johannes Krause, Tim Schüler, Shannon P. McPherron, Marcel Weiss, Jean-Jacques Hublin, Frido Welker

**Affiliations:** 1 Max Planck Institute for Evolutionary Anthropology, Leipzig, Germany; 2 School of Anthropology and Conservation, University of Kent, Kent, UK; 3 Chaire de Paléoanthropologie, CIRB (UMR 7241–U1050), Collège de France, Paris, France; 4 Department of Molecular and Cell Biology, University of California, Berkeley, Berkeley, CA USA; 5 Department of Evolutionary Genetics, Max Planck Institute for Evolutionary Anthropology, Leipzig, Germany; 6 Univ. Bordeaux, CNRS, Ministère de la Culture, PACEA, UMR 5199, Pessac, France; 7 Ancient Genomics Lab, Francis Crick Institute, London, UK; 8 Archaeological Micromorphology and Biomarker Lab, University of La Laguna, San Cristóbal de La Laguna, Spain; 9 Géosciences Environnement Toulouse (GET), Observatoire Midi-Pyrénées (OMP), Toulouse, France; 10 Department of Archaeology, School of Geosciences, University of Aberdeen, Aberdeen, Scotland; 11 Department of Anthropology, California State University Northridge, Northridge, CA USA; 12 Department of Evolutionary Anthropology, University of Vienna, Vienna, Austria; 13 Human Evolution and Archaeological Sciences (HEAS), University of Vienna, Vienna, Austria; 14 State Office for Heritage Management and Archaeology Saxony-Anhalt-State Museum of Prehistory, Halle, Germany; 15 Department of Archaeogenetics, Max Planck Institute for Evolutionary Anthropology, Leipzig, Germany; 16 Thuringian State Office for the Preservation of Historical Monuments and Archaeology, Weimar, Germany; 17 Department of Human Origins, Max Planck Institute for Evolutionary Anthropology, Leipzig, Germany; 18 Friedrich-Alexander-Universität Erlangen-Nürnberg, Institut für Ur- und Frühgeschichte, Erlangen, Germany; 19 Globe Institute, University of Copenhagen, Copenhagen, Denmark

**Keywords:** Archaeology, Biological anthropology

## Abstract

Recent excavations at Ranis (Germany) identified an early dispersal of *Homo sapiens* into the higher latitudes of Europe by 45,000 years ago. Here we integrate results from zooarchaeology, palaeoproteomics, sediment DNA and stable isotopes to characterize the ecology, subsistence and diet of these early *H. sapiens*. We assessed all bone remains (*n* = 1,754) from the 2016–2022 excavations through morphology (*n* = 1,218) or palaeoproteomics (zooarchaeology by mass spectrometry (*n* = 536) and species by proteome investigation (*n* = 212)). Dominant taxa include reindeer, cave bear, woolly rhinoceros and horse, indicating cold climatic conditions. Numerous carnivore modifications, alongside sparse cut-marked and burnt bones, illustrate a predominant use of the site by hibernating cave bears and denning hyaenas, coupled with a fluctuating human presence. Faunal diversity and high carnivore input were further supported by ancient mammalian DNA recovered from 26 sediment samples. Bulk collagen carbon and nitrogen stable isotope data from 52 animal and 10 human remains confirm a cold steppe/tundra setting and indicate a homogenous human diet based on large terrestrial mammals. This lower-density archaeological signature matches other Lincombian–Ranisian–Jerzmanowician sites and is best explained by expedient visits of short duration by small, mobile groups of pioneer *H. sapiens*.

## Abstract (web summary)

Using zooarchaeology, palaeoproteomics, ancient sediment DNA and stable isotope analyses, the authors characterize the ecology of the *Homo sapiens* individuals associated with the ‘transitional’ Lincombian–Ranisian–Jerzmanowician technocomplex at Ilsenhöhle in Ranis.

## Main

Reconstructing the ecological conditions and behavioural dynamics underlying the expansion of early groups of *Homo sapiens* into Eurasia is crucial to understand both the disappearance of Neanderthals and the global dispersal of our own species. Until recently, the earliest *H. sapiens* spreading across Europe were associated with the (Proto-)Aurignacian stone tool industry from circa 43 ka (thousand years ago) (cal BP)^1,2^. However, recent archaeological discoveries have provided direct evidence that early groups of *H. sapiens* were already present in Europe between 50 and 45 ka in Bulgaria (Bacho Kiro Cave)^3–5^, Czechia (Zlatý kůň)^6^ and Germany (Ranis)^7^, with preliminary claims from southeast France as far back as 54 ka^8^^,9^.

The expansion of *H. sapiens* into Europe has been linked to favourable climatic conditions during warm phases^10,11^, but recent stable isotope analyses indicate their presence during extreme cold climates^12,13^. This raises questions about the behavioural adaptations and survival strategies of these early *H. sapiens* populations. In-depth analyses of recovered faunal remains are limited, partly due to poor bone preservation^14–16^. In general, Upper Palaeolithic *H. sapiens* subsistence has been correlated with a shift in site use and occupation intensity and an expansion in diet breadth, to include larger proportions of smaller and faster animals, such as fish, birds, rabbits and foxes^14,17–20^. However, the subsistence strategies of *H. sapiens* groups during their first expansion onto the Northern European Plains 50–45 ka remain poorly understood.

**Fig. 1:**
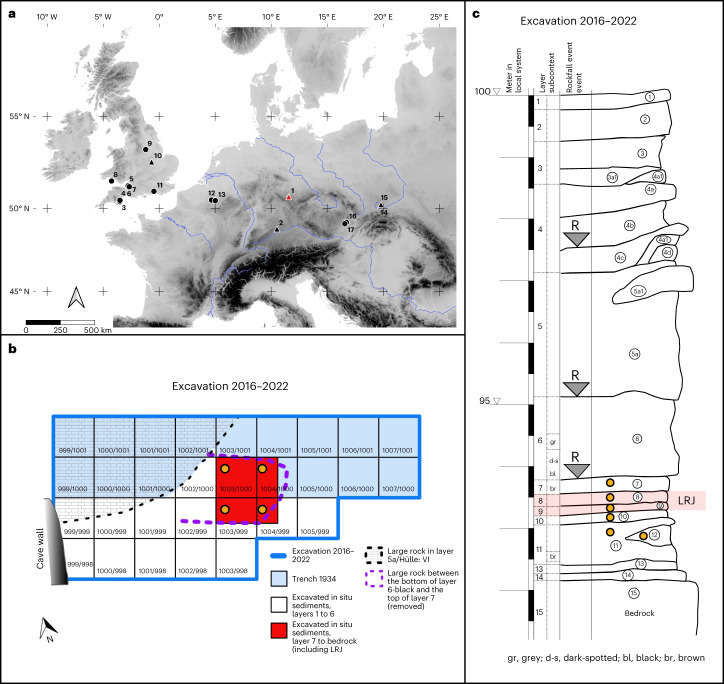
Geographic location, stratigraphy and excavation plan for the 2016-2022 excavations at Ranis. **a**, Geographic location of Ranis and the main LRJ sites, **b**, plan of the 2016–2022 excavations and **c**, stratigraphic sequence of the cave Ilsenhöhle at Ranis. Orange dots in **b** and **c** mark the layers and squares that were sampled for sedaDNA. R denotes rockfall events. See Mylopotamitaki et al.^7^ for the description of the sedimentary and chronological framework. In **a**, the location of main LRJ sites (1–7 and 9–15, adapted from Hussain et al^60^; 8, Aldhouse-Green^114^; 16–17, Demidenko and Škrdla^23^). Triangles mark sites with well-contextualized fauna. 1, Ranis; 2, Schmähingen-Kirchberghöhle; 3, Bench Quarry; 4, Kent’s Cavern; 5, Soldier’s Hole; 6, Hyena Den; 7, Badger Hole; 8, Paviland Cave; 9, Robin Hood’s Cave; 10, Grange Farm; 11, Beedings; 12, Spy; 13, Goyet; 14, Nietoperzowa Cave; 15, Koziarnia Green Cave; 16, Líšeň Podolí I; 17, Želešice III. The map was created in QGIS based on Shuttle Radar Topography Mission data V4 (http://srtm.csi.cgiar.org)^116^. In **b**, Each numbered square is 1 m^2^. The basal sequence including the LRJ layers was excavated in the red area of squares 1003/999, 1003/1000, 1004/999 and 1004/1000. Panels **a** and **b** were created with Affinity Designer version 2.3.0.2165.

Recent excavations (2016–2022) at the cave Ilsenhöhle in Ranis (hereafter Ranis, Thuringia, central Germany; Fig. 1) have yielded well-preserved faunal assemblages across its stratigraphic sequence, which includes layers with non-diagnostic tools (layers 12–11)^7,21^, the Lincombian–Ranisian–Jerzmanowician^21–23^ (LRJ, layers 9–8) and the Upper Palaeolithic (layers 6–4a; Fig. 1). The main focus of this paper is on fauna from these excavations and more specifically LRJ layers 9 and 8, which have been dated to 47,500–45,770 cal BP and 46,820–43,260 cal BP, respectively^7^. These layers are associated with multiple skeletal remains of *H. sapiens*^7^.

For further contextualization, we conducted detailed analyses of the overlying layer 7 and underlying layers 12–10. To enlarge the faunal reference baseline for the isotopic analysis, we also include stable isotope data from faunal remains from the 1932–1938 excavations, including directly radiocarbon dated equid remains that are equivalent in age with layer 7 (2016–2022 excavations) or older^13^ and faunal material recovered from layer IX^21^. We applied a multidisciplinary approach, integrating methods from zooarchaeology, palaeoproteomics, sediment DNA and bulk stable isotopes (Supplementary Table 1). The integration of these different datasets allows for a detailed reconstruction of the animal species present at the site ~45 ka, their accumulation agents, food webs and human subsistence practices. We propose a model in which the ephemeral involvement of early *H. sapiens* with the faunal accumulation at Ranis can be related either to small group sizes or short site visits by highly mobile human groups.

## Results

### Bone fragment identification

We analysed a total of 1,754 piece plotted remains and using traditional comparative morphology were able to taxonomically identify 9.7% (*n* = 170), consistent with other Late Pleistocene sites^14,19^. Zooarchaeology by mass spectrometry (ZooMS; *n* = 536) provided additional taxonomic identifications to either family or species level for over 98% of the analysed specimens (*n* = 530; 98.9%; AmBic extractions). This increased our overall identification rate to 40% (*n* = 700). The LRJ fauna is dominated by cervids (layer 8 = 36%, layer 9 = 29%; Supplementary Table 2) that are mainly reindeer (*Rangifer tarandus*), although red deer (*Cervus elaphus*) are present as well. Other large herbivores, such as equids (layer 8 = 8%, layer 9 = 9%) and bovids (layer 8 = 8%, layer 9 = 11%) occur in lower proportions. Furthermore, there is a high percentage of Ursidae (mainly *Ursus speleaus*, layer 8 = 28%; layer 9 = 29%), and carnivores (3.5–7.5%) from a broad range of taxa (Canidae, Hyaenidae/Pantherinae, Felinae, red fox (*Vulpes vulpes*), Arctic fox (*Vulpes lagopus*) and wolverine (*Gulo gulo*)) are present in low numbers. ZooMS identified Elephantidae (most likely *Mammuthus primigenius*) and Rhinocerotidae (most likely *Coelodonta antiquitatis*), which were absent in the morphologically identifiable fraction. We also applied species by proteome investigation (SPIN) to all the morphologically unidentifiable fauna from layer 8 (*n* = 212), which confirmed the identifications made through ZooMS. SPIN was able to provide additional taxonomic resolution for 10 of the ZooMS samples, specifying them as *Bison* sp. (Supplementary Table 7 in Mylopotamitaki et al.^7^). Overall, the identified fauna is representative of a marine isotope stage 3 cold-stage climate with a largely open tundra-like landscape^7,13^.

**Fig. 2:**
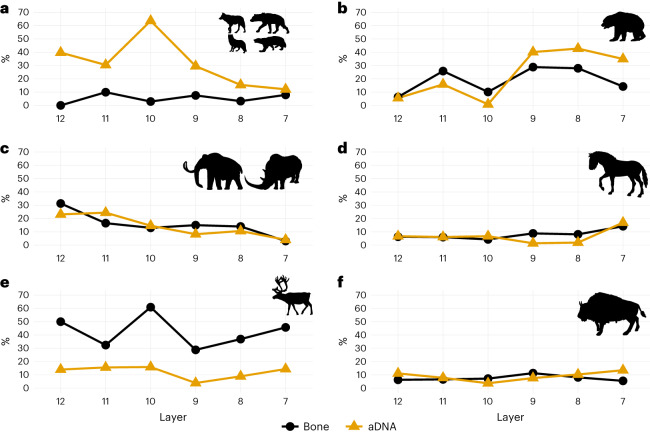
Overview of the bone fragments and ancient mammalian DNA identified across layers 12–7 at Ilsenhöhle in Ranis. The bone fragment line includes identifications both based on morphology and through ZooMS (for a breakdown by method, see Extended Data Table 1). **a**, Carnivores, including Canidae, Hyenaidae, Felinae, red fox (*V. vulpes*) and wolverine (*G. gulo*). **b**, Ursidae (*Ursus spelaeus*, *Ursus arctos*, *Ursus* sp.). **c**, Megafauna, including mammoth (*M. primigenius)* and rhinoceros (*C. antiquitatis*). **d**, Equidae (*Equus ferus* and *Equus* sp.). **e**, Cervidae, including reindeer (*R. tarandu*s) and *Cervus* sp. **f**, Bovidae (*Bos primigenius*, *Bison priscus*, *Bos*/*Bison*). The proportion of aDNA was calculated based on the number of ancient mtDNA fragments assigned to each taxon per layer (Supplementary Tables 3–6). The % on the *y* axis includes %NISP for bone fragments and percentage of identified sedaDNA (%sedaDNA). Animal silhouettes downloaded from https://www.phylopic.org/.

The faunal spectrum of layers 9–8 is largely consistent with the overlying layer 7 and the underlying layers 12–10 (Fig. 2), although sample sizes are variable (Supplementary Table 2). In general, there is a decrease in megafauna (mammoth and rhinoceros) and an increase in ursids forward through time, while the proportion of equids and bovids remains relatively stable (Fig. 2). Layer 10 is marked by an increase in reindeer and a lower abundance of carnivore and ursid bones. To assess whether the change in the proportion of these NISP (number of identified specimens) values between layers was statistically significant, we calculated composite chi-square values and adjusted residuals (Extended Data Table 1). There were significant differences in taxonomic proportions. Between layers 11 and 10 this was driven by an increase in Cervidae remains and a decrease in Ursidae remains. Between layers 10 and 9 this pattern was reversed (Fig. 2). For layers 8–7 the differences are driven by notable increases in carnivore remains and larger herbivores, including equids and cervids, while the proportion of both Ursidae and megafaunal remains is reduced significantly.

### Species diversity and taxonomic richness

**Fig. 3:**
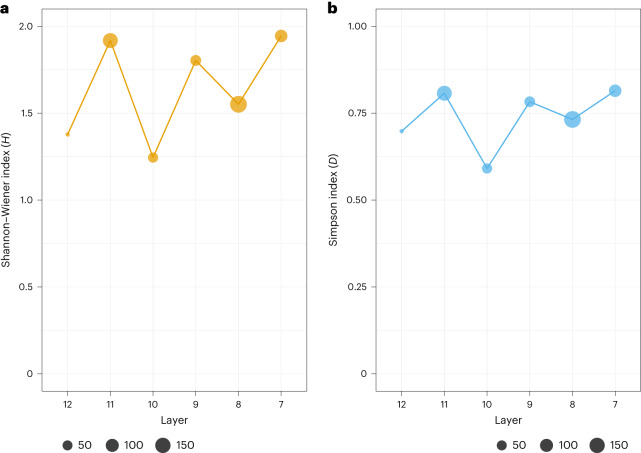
Ecological diversity indices for faunal assemblages from layers 12–7 at Ranis. **a**, Shannon–Wiener index, which measures faunal diversity (*H*), and **b**, Simpson’s assemblage evenness index (*D*) show respective increased diversity and assemblage evenness per layer; values for *H* are normally between 1.5 and 3.5, with larger values indicating greater taxonomic diversity; values for *D* are between 0 (no taxonomic evenness) and 1 (complete taxonomic evenness); note that different scales are used on the *y* axis in **a** and **b**; see Supplementary Table 7 for a breakdown of NISP, NTAXA and ecological indices. Point size is scaled to the NISP.

There is a relatively high number of taxa (NTAXA) in all layers (5 to 12 per layer; Fig. 3 and Supplementary Table 3) identified through both comparative morphology and ZooMS analysis. In general, NTAXA and taxonomic richness are positively correlated with sample size, and this is also true at Ranis^24–26^. For example, the lower NTAXA in layer 12 (NTAXA = 5) can be explained by the small number of bone fragments recovered from this layer (*n* = 18). We see some variation in faunal diversity through layers 12–7 reflected by fluctuations in the Shannon–Wiener and Simpson’s indices (Fig. 3), which are used to measure faunal diversity^24^. At Ranis we see higher values for these diversity indices in those layers with the highest proportions of carnivore modified remains (layers 11, 9 and 7). In fact, despite layers 11 and 8 having similar assemblage sizes, taxonomic diversity and assemblage evenness are different, with lower values for layer 8.

### Ancient sediment DNA

**Extended Data Fig. 1:**
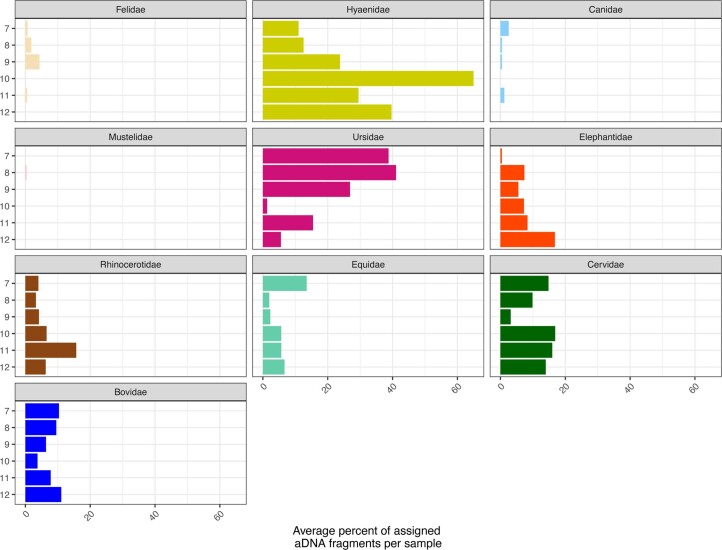
Mammalian ancient DNA recovered from sediment samples from the 2016-2022 excavations at Ilsenhöhle in Ranis. 26 sediment samples were analysed across Layers 7 (n samples = 5), 8 (n samples = 6), 9 (n samples = 3), 10 (n samples = 2), 11 (n samples = 9) and 12 (n samples = 1). Assignments to Cricetidae are not included in this figure. A detailed breakdown of the aDNA data can be found in SI Table 5.

Twenty-six sediment samples were collected from layers 12–7 (Fig. 1 and Supplementary Tables 4–7) to test for the preservation of ancient mammalian DNA. All 26 samples contained evidence for the presence of ancient mammalian DNA, with between 4,991 and 63,966 unique mammalian mitochondrial DNA sequences recovered from each sample. These sequences were assigned to a total of 11 mammalian families, each of which was represented by between 1,416 and 15,631 sequences (Extended Data Fig. 1). Ancient Bovidae, Cervidae, Elephantidae, Equidae, Hyaenidae, Rhinocerotidae and Ursidae DNA was recovered from all layers (Fig. 2). As has been seen in other sediment DNA studies, more large than small mammals were identified^27^. The proportion of DNA fragments recovered from a given taxon is not necessarily expected to correlate strongly with the proportion of bone fragments due to differences in taphonomy, body mass, activity among species at sites, laboratory processes (for example, hybridization capture design) and sequence identification. However, as trends for the relative amount of DNA or skeletal remains of large mammals have been previously shown to be complementary^27^, we calculated the average proportion of mtDNA fragments assigned to each family per sediment sample per layer to investigate this relationship in a different location. At Ranis the ancient sediment DNA (sedaDNA) and bone fragment data follow similar patterns (Fig. 2), with a decrease in megafauna towards the younger layers coupled with an increase in Ursidae. While the relative amount of Bovidae and Cervidae DNA was consistent throughout the layers, the proportion of carnivore (especially Hyaenidae) DNA is more variable (Fig. 2). In layer 10 this increase in Hyaenidae DNA correlates with a peak in Cervidae bone fragments, a decrease in carnivore bone fragments and an increase in hyaena coprolites as seen at other Pleistocene sites^28,29^. Overall, the consistency between the identified taxa in the sedaDNA and the zooarchaeological records confirms the previous notion that sedaDNA analysis can provide a relatively quick and simple method for assessing, at least broadly, the past diversity of large mammals at caves with DNA preservation.

### Find densities

During the 2016–2022 excavations 1,754 bone piece-plotted remains (>20 mm) and 76 lithic remains (mostly <20 mm) were recovered from layers 12–7 (Extended Data Table 2), with higher densities in layers 9–7 and especially within layer 8 (bone density = 1.44; lithic density = 0.23). By contrast, the sedaDNA density (number of sequences identified per milligram of sediment) is highest in layers 12–11, while there is a twofold to threefold decrease in ancient animal sequences within LRJ layers 9–8 (Extended Data Table 2). It should be noted that the DNA libraries used for this analysis were not sequenced to exhaustion (see duplication rates in Supplementary Table 6) and that deeper sequencing may change these results. In addition, differences in the geochemistry between layers may impact the DNA preservation and resulting density calculations. Taken together, the density of lithic, bone and ancient DNA suggests a complex picture of site use. The most intense use of the site by *H. sapiens* occurs in layer 8, while the input of human groups in other layers appears even more ephemeral with the site potentially used more extensively and over a longer time by larger carnivores (Fig. 2).

### Bone fragmentation and preservation

**Extended Data Fig. 2:**
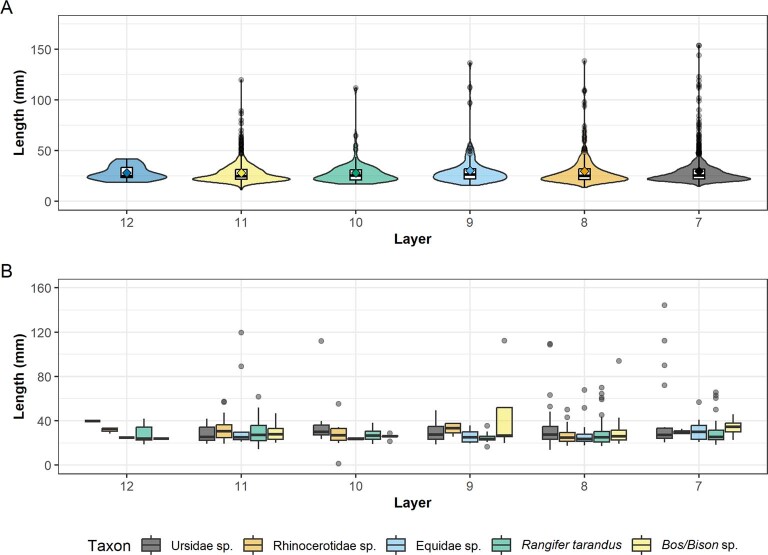
Overview of the length of all piece-plotted bone fragments recovered across Layers 12-7 at Ilsenhöhle in Ranis; B: Overview of length for major taxa from Layers 12-7 at Ilsenhöhle in Ranis. Figure 2a sample sizes are: Layer 12 (n = 18); Layer 11 (n = 565); Layer 10 (n = 92); Layer 9 (n = 115); Layer 8 (n = 244); Layer 7 (n = 722). Figure 2b sample sizes are: Ursidae (Layer 12 n = 1; Layer 11 n = 47; Layer 10 n = 7; Layer 9 n = 23; Layer 8 n = 62; Layer 7 n = 18); Rhinocerotidae sp. (Layer 12 n = 3; Layer 11 n = 13; Layer 10 n = 8; Layer 9 n = 5; Layer 8 n = 28; Layer 7 n = 3); Equidae sp. (Layer 12 n = 1; Layer 11 n = 11; Layer 10 n = 3; Layer 9 n = 7; Layer 8 n = 18; Layer 7 n = 18); Rangifer tarandus (Layer 12 n = 6; Layer 11 n = 32; Layer 10 n = 32; Layer 9 n = 13; Layer 8 n = 67; Layer 7 n = 27); Bos/Bison sp. (Layer 12 n = 1; Layer 11 n = 12; Layer 10 n = 5; Layer 9 n = 9; Layer 8 n = 18; Layer 7 n = 7). Box plot in Extended Data Figs. 2a and 2b: box extends from first quartile (Q1 on left) to third quartile (Q3 on right) with bold line in middle representing (median); Lines extending from both ends of the box indicate variability outside Q1 and Q3; minimum/maximum whisker values are calculated as Q1/Q3 -/ + 1.5 * IQR. Everything outside is represented as an outlier.

Piece-plotted bone remains are similarly fragmented across layers 12–7 with a majority between 25 mm and 50 mm long and a small number of pieces larger than 100 mm (Extended Data Fig. 2 and Supplementary Table 8). A *t*-test shows no significant difference between the layers (Supplementary Table 9). The major taxa from layers 12–7 are similarly fragmented with comparable average bone length (Extended Data Fig. 2 and Supplementary Tables 10–13) and statistical tests illustrate no significant difference between either dominant taxa or between major taxa within these layers. Overall, extensive bone assemblage fragmentation prevents further discussions of either skeletal representation or transport decisions (see Supplementary Table 14 for data on zooarchaeological quantification including NISP, minimum number of elements (MNE) and minimum number of individuals (MNI)).

**Fig. 4:**
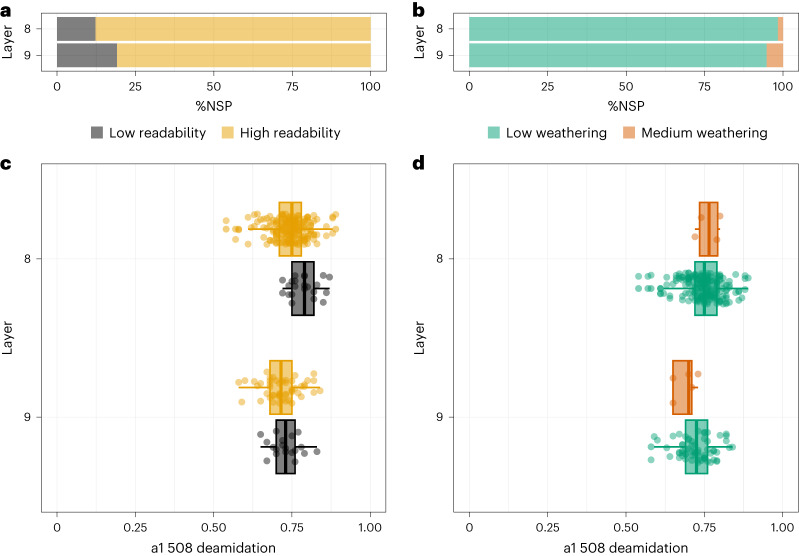
Bone preservation at Ranis in LRJ layers 8 and 9. **a**, Bone surface readability by layer; low readability (0–50% bone surface remaining), high readability (51–100% bone surface remaining), see Methods for further details. **b**, Bone weathering by layer; low weathering (stages 0 and 1); medium weathering (stages 2 and 3) based on Behrensmeyer^71^. **c**, COL1ɑ1 508–519 deamidation by layer plotted with bone readability. **d**, COL1ɑ1 508–519 deamidation by layer plotted with bone weathering stages. Sample sizes in **c**: layer 8: low readability (*n* = 22), high readability (*n* = 168); layer 9: low readability (*n* = 19), high readability (*n* = 50). Sample sizes in **d**: layer 8: low weathering (*n* = 186), high weathering (*n* = 4); layer 9: low weathering (*n* = 64), high weathering (*n* = 5). Box plots in **c** and **d**, box extends from first quartile (Q1 on the left) to third quartile (Q3 on the right) with bold line in the middle representing the median. Lines extending from both ends of the box indicate variability outside Q1 and Q3; minimum/maximum whisker values are calculated as Q1/Q3 ± 1.5 × IQR. Everything outside is represented as an outlier. IQR, interquartile range.

**Extended Data Fig. 3:**
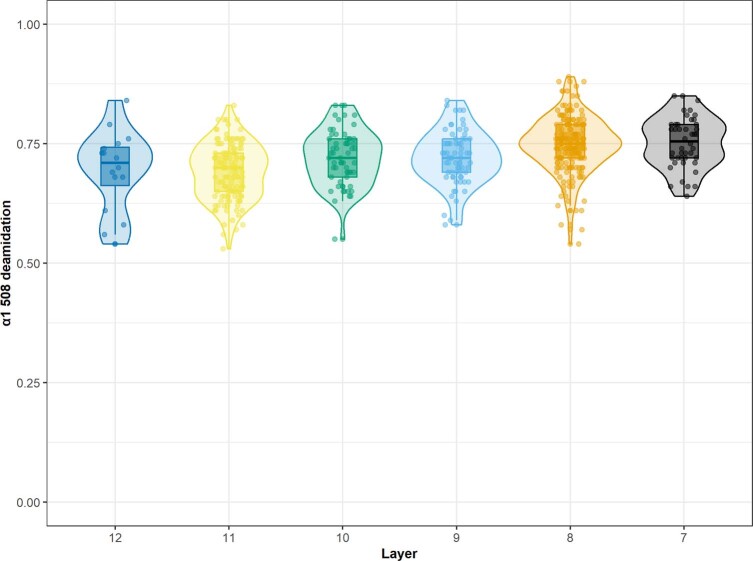
Glutamine deamidation values for α1 508 of the bone fragments analysed through ZooMS. These are seen as an indicator for the biomolecular preservation of the bone. Sample sizes are Layer 7 (n = 44), Layer 8 (n = 190), Layer 9 (n = 69), Layer 10 (n = 61), Layer 11 (n = 133) and Layer 12 (n = 16); see SI Tables 14 and SI Table 15. Box plot in Extended Data Fig. 3: box extends from first quartile (Q1 on left) to third quartile (Q3 on right) with bold line in middle representing (median); Lines extending from both ends of the box indicate variability outside Q1 and Q3; minimum/maximum whisker values are calculated as Q1/Q3 -/ + 1.5 * IQR. Everything outside is represented as an outlier.

Bone fragments from all layers are well preserved with a high percentage of original bone surface remaining and low percentage of sub-aerial weathering (Fig. 4 and Extended Data Table 3). Biomolecular preservation was assessed through the calculation of glutamine deamidation values, which are indicative of protein preservation^30^. Deamidation values were obtained for 518 of the bone fragments that were part of the ZooMS analysis (97%). The deamidation values for COL1ɑ1 508–519 cluster between 0.60 and 0.80 (Extended Data Fig. 3 and Supplementary Tables 15 and 16). No outliers are present, which could represent intrusions into the archaeological unit or differential bone preservation. A comparison across layers shows that deamidation values largely overlap, with a slight trend towards lower values (thus poorer preservation) deeper down the stratigraphic sequence. Wilcoxon tests illustrated significant differences in deamidation between layers (especially between layers 7 and 11 and between layers 8 and 11) (Supplementary Table 17). This difference, though, could relate to variations in sample sizes. A Wilcoxon test showed there were no significant differences in COL1ɑ1 508–519 deamidation values by bone fragment size (Supplementary Table 18). Overall, despite their high fragmentation, the LRJ bone fragments are well preserved and show neither difference in macroscopic alterations nor biomolecular preservation, indicating a consistent diagenesis.

### Bone surface modifications

**Fig. 5:**
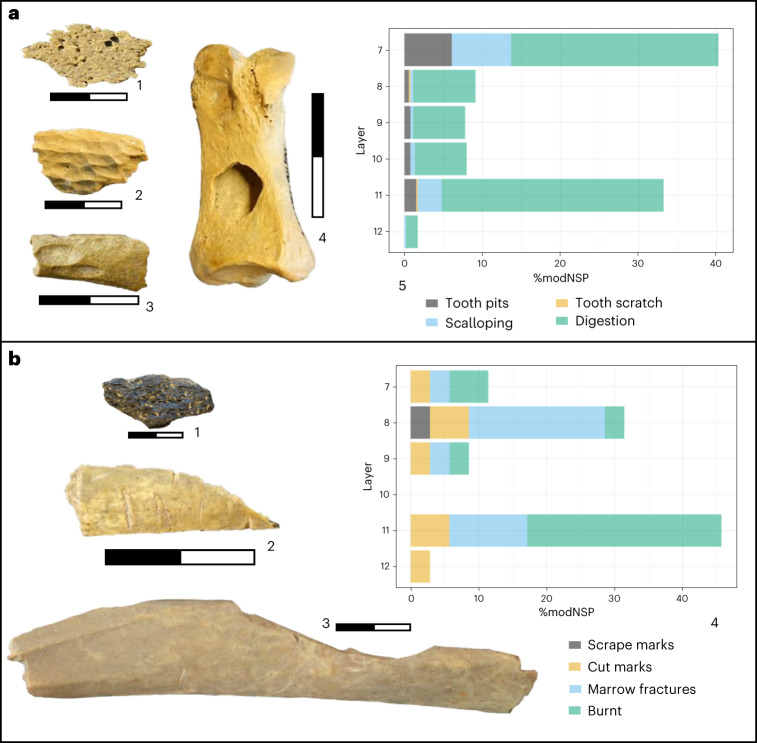
Bone surface modifications from Ranis. **a**, Carnivore modifications: 1, digested piece (layer 10, 16/116-159503); 2, digested piece (layer 7, 16/116-151389); 3, carnivore gnawing (layer 8, 16/116-151583); 4, *U. spelaeus*: carnivore tooth pit (layer 11, 16/116-186374); 5, graph illustrates different carnivore modifications as percentage of total number of carnivore modifications (*n* = 527) across all layers (%modNSP). **b**, Human modifications: 1, unknown mammal: burnt fragment (layer 11, 16/116-186401); 2, cut-marked fragment (layer 9, 16/116-159345); 3, *C. elaphus*: marrow fracture (layer 8, 16/116-159070); 4, graph illustrates different human modifications as percentage of total number of human modifications (*n* = 35) across all layers (%modNSP); for data underlying both graphs, see Supplementary Table 21. All photo scales are in centimetres.

Across all layers carnivore modifications are abundant and dominant, ranging from 19% to 44%, across a range of species, including rhinoceros, reindeer, bovids and equids. This includes traces of gnawing (tooth pits, scalloping and scratches) and digestion (acid etching; Fig. 5). Carnivore modifications are highest in layers 7 and 10, which also preserve coprolite material (Supplementary Fig. 1). Micromorphological analysis of one coprolite (sample 116 159507, layer 7) indicates a carnivore origin, possibly hyena or canid (Supplementary Fig. 1), and further detailed analyses are ongoing.

Human modifications, including marrow fractures and cut marks (Fig. 5), are very sparse in layers 9–8 (3.5–4.1%) and 12–11 (3.0–5.6%) and (near) absent in layers 7 (0.6%) and 10 (0.0%) (Supplementary Table 19). We calculated a chi-square test with adjusted residuals to assess whether the proportion of human and carnivore bone surface modifications showed significant differences between all layers. There was a statistically significant difference between layers 7 and 8 (*χ*^2^ = 14.9, *P* = <0.01) driven by an increase in the proportion of carnivore modified bones compared to human modifications (Supplementary Table 20).

LRJ layers 9–8 have the highest proportion of bones with human butchery modifications and the lowest proportion of carnivore modifications, although these are still high and predominant (Supplementary Tables 19 and 21). Anthropogenic modifications throughout layers 12–7 are predominantly represented by marrow fractured elements of a range of large ungulates, including Equidae and Cervidae (Extended Data Table 4) with limited evidence for meat removal on mammal long bones. We identified limited exploitation of carnivores at Ranis with a cut-marked red fox (*V. vulpes*) mandible from layer 8 and a cut-marked wolf (*Canis lupus*) mandible from layer 11. Furthermore, we identified a single cut-marked bird bone in layer 8, suggesting the limited exploitation of avian taxa.

Among the faunal fragments larger than 20 mm, only 14 show macroscopic evidence for burning (Fig. 5). These burnt fragments show a range of temperature-induced colour changes from carbonized (stage 1) to fully calcined (stage 5), and despite a concentration in layer 11 (64.3%; *n* = 9), the overall low quantity of burnt material prevents further analysis of spatial or temporal trends.

### Seasonality and site use

Only 21 post-cranial fragments from layers 12–7 are fetal, unfused or with incomplete element fusion, providing limited data on biological age, with most of the elements representing adult individuals. Dental remains, especially the presence of deciduous dentition and unerupted molar teeth, provide seasonality data from most layers at Ranis for both carnivore and herbivore taxa (Supplementary Table 22). The pattern of seasonality in all layers at Ranis, including the main LRJ layers 9–8, suggests animals died during all seasons of the year but especially during the spring and summer months (March to August). The low anthropogenic signal at Ranis means that such seasonality indicators most probably relate to carnivores rather than human occupation at the site. Further analysis of dental fragments from the screened residues could help to further clarify these seasonality patterns.

Ursidae remains provide the most seasonality information (Methods), although only from layers 8 and 7. We identified mainly juvenile individuals (layer 7, *n* = 3; layer 8, *n* = 3) and a single prime-aged individual from layer 7 (Supplementary Table 22). Eruption and wear stages of the Ursidae teeth (I–III) suggest young individuals (some potentially between 5 and 12 months old) that died toward the end of hibernation (late winter to spring)^31,32^. Other individuals suggest they died during spring and summer months after leaving hibernation. Finally, the presence of an unerupted manidublar molar 3 (M_3_) indicates an individual that died, perhaps, during its second hibernation. The low quantity of human modifications on these cave bear remains suggests that most of these represent natural deaths during hibernation.

### Diet and ecology

**Fig. 6:**
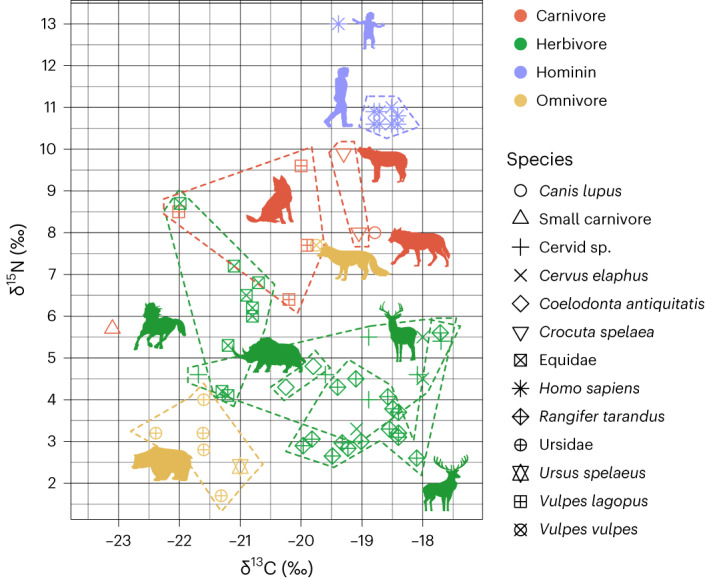
Bulk collagen *δ*^13^C and *δ*^15^N values for mammal remains from layers 12–7 (2016–2022 excavations) and layers XI–VIII from the 1932–1938 excavation at Ranis. Mammalian isotope data: *n* = 52. Hominin isotope data: *n* = 10.

Mammalian isotope data (*n* = 52) reveal niche separation between species (Fig. 6 and Extended Data Table 5). Comparatively high *δ*^13^C values are consistent with lichen consumption in cervid species^33,34^, especially reindeer (*R. tarandus*), and (isotopic) niche separation from equids is clear during the colder phase between ~45 and 43 ka cal BP^13^ (Fig. 6). Cave bear remains from layers 7 and 9 have low *δ*^15^N values typical of this species, consistent with an herbivorous diet^35^. Carnivore remains of foxes (*V. vulpes and Alopex lagopus*), wolves and hyaenas show higher *δ*^13^C and *δ*^15^N values consistent with their anticipated trophic level. The absence of *δ*^13^C values lower than −22.5‰ in any herbivore species indicates an open environment or lack of woodland cover^36,37^ (Supplementary Figs. 2 and 3). Combined with prevalent lichen consumption by cervids, this is consistent with other stable isotope data from the site, showing that the LRJ occupation of Ranis took place in a cold steppe or tundra setting^13^.

Similar *δ*^13^C values for *H. sapiens* and herbivores suggests humans consumed a range of terrestrial mammal species, including horse, rhinos and reindeer. Nitrogen isotope ratios for the Ranis *H. sapiens* are more consistent with Neanderthals^38,39^ than with early Upper Palaeolithic *H. sapiens* (Supplementary Fig. 4a, Extended Data Table 5 and Supplementary Tables 23 and 24). However, taking into account the isotope ratios observed in the associated fauna, the trophic level enrichment looks similar to that of their Goyet Neanderthal contemporaries, as well as the later *H. sapiens* from Buran Kaya and Kostenki^40,41^ (Supplementary Fig. 4b,e). This suggests that Ranis *H. sapiens* mainly relied on similar resources as those individuals, that is, terrestrial animals, for their protein intake and no (or small amounts of) aquatic foods^41,42^. It supports the hypothesis of Bocherens et al.^41,43^ that different nitrogen isotope ratios between Upper Palaeolithic *H. sapiens* and Neanderthals are not related to different subsistence strategies between the two species but are related to a change of baseline over time (Supplementary Fig. 4c,d). When comparing the average *δ*^15^N values of the humans and associated herbivores, humans show higher values beyond what could be expected for a diet based on these species (that is, 7% as opposed to the 3–5% typical of trophic level enrichment). For Goyet and Buran Kaya, it has been interpreted as a sign of frequent mammoth meat consumption^41,42^. We did not obtain any nitrogen isotope ratios from mammoth remains in Ranis, and other species that typically show high *δ*^15^N values (for example, freshwater fish) were not found at the site (Supplementary Information). However, woolly rhinos and horses show high *δ*^15^N values compared to other local herbivores. Their consumption, or consumption of other foods with high *δ*^15^N, possibly from sites occupied in other times of the year, could therefore explain the high human *δ*^15^N values.

The diet of the ten *H. sapiens* fragments studied is remarkably homogeneous, with all samples but one being within 1‰ of each other. The mtDNA^7^ suggests a minimum of six individuals, indicating that inter-individual dietary variability was low with a relatively stable resource base during the different periods of site occupation. By contrast the human individual R10874 has higher *δ*^15^N values (by ~2–2.5‰), which is close to the range of typical trophic level enrichment (3–5%). Based on morphological characteristics of the bone specimen, this individual appears to be a juvenile, and further assessment is ongoing^44,45^.

## Discussion and conclusion

*H. sapiens* expanded into the higher latitudes of Europe by 45 ka^7^. Our multi-proxy approach indicates that between 55 and 40 ka (layers 12–7) the large cave Ilsenhöhle at Ranis was predominantly used for hyaena denning and cave bear hibernation. In general, carnivore dens contain a higher species diversity compared to human accumulations^46^, and we have illustrated the important role of carnivores in the faunal accumulation in the LRJ layers at Ranis. Human presence fluctuated as seen by the presence of morphologically identifiable human remains, humanly modified bones and stone artefacts^7^. *H. sapiens* occupation occurred initially during climatic conditions ~7–8 °C cooler than today (~48–45 ka), followed by their presence during a period of extreme cold^13^ (~45–43 ka), as indicated by abundant cold-adapted taxa (for example, reindeer, wolverine, arctic fox, woolly rhino and mammoth) and stable isotope data. Traces of fire use are sparse, although micromorphological analysis does indicate increased fire use in layer 8^7^ compared to other layers at Ranis. Human butchery signatures are scarce and mainly focused on marrow exploitation from a range of species (equids, cervids and, occasionally, carnivores). Stable isotope data confirms a human diet focused on cervids (including reindeer), rhinoceros and horse with *δ*^*1*3^C and *δ*^15^N values suggesting these early *H. sapiens* populations had a diet similar to contemporary Neanderthals. The significant enrichment in *δ*^15^N levels in juvenile R10874 suggests that breast milk was the primary source of dietary protein. However, the low *δ*^13^C value for this individual, compared to others, cannot be explained by breast milk consumption alone. This low carbon value could be consistent with breast milk consumption if the nursing person had a diet including more horse meat than others or if the juvenile individual was weaned but experienced a prolonged period of catabolic stress before their death^44,45,47,48^.

While LRJ leaf points have been found at over 40 find spots across the Northern European Plains^22^, reconstructions of LRJ human subsistence behaviour are limited as much of the material originates from either older (and often poorly contextualized, recorded and/or dated) excavations or sites with poor bone preservation (for example, Beedings, UK^49^; Extended Data Table 6). In recent years, several new LRJ excavations and up-to-date reassessments of old collections^23,42,50–52^ have been undertaken. These indicate that despite its large geographic extension, from Moravia into Britain, LRJ occupations predominantly relate to cold, open environments with grassland and shrub tundra comprising juniper, dwarf birch and willow^52–57^. At LRJ sites cold-adapted species dominate (for example, horse, woolly mammoth, woolly rhinoceros, reindeer and lemming), and carnivores (for example, wolf, hyaena and red fox) played a dominant role in the accumulation of the faunal remains, as indicated by a high frequency of gnawing marks, carnivore skeletal part profiles dominated by teeth^51,52,58,59^ and at Ranis an increase in hyaena sedaDNA. Conversely, human input at LRJ sites is generally low, and this ephemeral presence of human activity in carnivore dens is a common feature across the Palaeolithic, including in Middle Palaeolithic and Châtelperronian contexts^60,61^.

Combined with low artefact densities and scarce fire use, we suggest a low-intensity site use by these early groups of *H. sapiens* and an LRJ settlement pattern dominated by short-term hunting stations^23^. This low archaeological signature contrasts with the Initial Upper Palaeolithic *H. sapiens* occupation at Bacho Kiro Cave where we see an increasingly intense use of the site (including fire) alongside the specialized exploitation of carnivore carcasses and the use of bone as raw material for tools and ornaments^14,62^. The scarce archaeological signature of the LRJ can be best explained by small group sizes of these pioneer *H. sapiens* populations. Their highly mobile lifestyles resulted in expedient visits of short duration at localities which are otherwise occupied by carnivores. The presence of a sub-adult individual opens up the possibility that these short-term stays included family groups, although further osteometric and nuclear DNA data from all Ranis individuals is needed to clarify these patterns. Additional excavations of well-contextualized LRJ sites with good bone preservation will be key to understand fully the variability within the ecology, diet and subsistence of LRJ *H. sapiens* during their dispersal across the higher latitudes of Europe.

## Methods

A total of 1,754 piece-plotted remains were analysed through a combination of traditional and biomolecular approaches. This includes all material from the lower layers of the new excavation (layers 12–7; Supplementary Table 1). In general, an untargeted sampling strategy was used to select morphologically unidentifiable bone for ZooMS analysis throughout layers 12–7. The importance of layers 8 and 9 for identifying and understanding the makers of the LRJ meant that all unidentifiable bone remains were sampled through ZooMS and a majority analysed through SPIN. A fragment size cut-off of bone length >20 mm was used to ensure that taxonomically identified fragments could be subjected to further biomolecular analyses in the future, if needed. Overall, 30.7% of the total bone remains from layers 12–7 were analysed with ZooMS. A detailed description and account of the excavation strategy, sedimentary analysis, micromorphology and lithics are provided in Mylopotamitaki et al.^7^.

### Zooarchaeology

All faunal material from layers 12–7 was studied using traditional comparative morphological approaches. The faunal reference collection stored at the Max Planck Institute for Evolutionary Anthropology (Leipzig) alongside reference atlases were used to assign fragments to species and skeletal elements, where possible^63,64^. To understand site use and human behaviour at Ranis, a series of taphonomic attributes were recorded on each bone and combined with specific taxon, body part identifications and where applicable various indices of zooarchaeological quantification including MNE, MNI and minimum anatomical units (MAU). The NISP value is the number of specimens identified to species and element^65,66^; when an accurate taxonomic identification was unclear, fragments were recorded to the family level (for example, Ursidae species) or specific body size class (for example, ungulate large; based on Morin^18^ and Smith et al.^14^). The MNE was calculated by selecting the zone with the highest representation of >50% present, which was further combined with side and fusion data for each specific element^14,67–70^. The MNI was calculated for each specific element (including left and right) with an overall value for each taxon chosen by selecting the highest value.

All bone fragments were studied under magnification (×20) using an oblique light source, to assess bone surface preservation and the presence of specific bone surface modifications. The proportion of original bone surface remaining was recorded and expressed as a percentage ranging from 0% (no original surface remains) to 100% (all bone surface remaining)^14^. We recorded bone surface weathering using Behrensmeyer^71^, which provides a qualitative scale for understanding the exposure (short/long) of bone material before deposition. Root etching and abrasion (expressed as a percentage of bone surface affected) were recorded and range from 0% (no visible modification observed) to 100% (the whole bone surface covered^14,67,68^). We used Stiner et al.^69^ to record the specific colour and surface changes associated with burning and fire use.

Specific carnivore modifications recorded included tooth pits, scratches, crenelation and damage from digestion^14,66,67,70^. Human modifications included those related to butchery and carcass processing such as cut marks, skinning marks and deliberate marrow fractures (identification of impact point and/or percussion notches^66,70^), alongside other secondary uses of organic material for informal bone tools (‘retoucher’), formal bone tools (lissoirs, awls and so on) and ornaments^3,62^.

We calculated ecological diversity indices to investigate the diversity of the faunal community within layers 12–7 at Ranis. We calculated the Shannon–Wiener index (H′)^72,73^ to quantify the taxonomic diversity of the faunal assemblages (which combined morphologically and ZooMS-identified specimens). The Shannon–Wiener index is sensitive to sample size, so some values should be evaluated with caution when sample size is small^72^. This index produces values that typically range between 1.5 and 3.5 with larger values indicating taxonomic heterogeneity^72^. The Simpson’s index of evenness provides a bias-adjusted estimate of evenness in the population from which sub-samples are derived and studied. This makes it a more preferred method for measuring evenness^72^. The index value ranges from 0 (no taxonomic evenness) to 1 (complete taxonomic evenness). In short, the closer the calculated value for the Simpson index is to 1 then the more that assemblage is dominated by a single taxon^72^.

Age and seasonality indicators were calculated from various species using both cranial (mainly teeth eruption and wear) and postcranial bone fusion data^73^. Herbivore age was calculated using various methods depending on tooth type. For species with low-crowned teeth such as *Bos*, *Bison* and cervids, the quadratic crown height measure was applied^74–76^ along with established wear stages^77^. For equids, crown height was measured on juveniles and adults and calculated using established equations^78,79^ and tooth wear stages documented^80^. Bear dentition was scored according to the three-stage scheme devised by Stiner^31,32,81–84^. Bears have an unusual dental development and eruption, as they are born during hibernation (winter, January), compared to other carnivores (hyena and canids) and ungulates (generally spring time, late May)^81,85^. All bears are born during hibernation (peak time January) and are toothless, although full deciduous dentition emerges by the third month with the permanent first molar (M1) usually by the fifth month. Bears generally have all permanent dentition erupted by the end of the first year with the eruption of the permanent canines starting during the second year and completion by the end of the third year of life. Using specific timing and eruption of deciduous and permanent dentition allows for the development of a tooth eruption wear scheme that includes nine stages, grouped into three age categories (juvenile (I–III), prime (IV–VII) and old (VIII–IX))^31,32,82^. Although the scheme does not provide an estimate for the age at death, it provides the ability for intersite and intrasite comparisons at an ordinal scale^31^.

All analyses were undertaken in R, v. 4.3.2^86^ using RStudio, v. 2023.03.1^87^, mainly by using the ‘tidyverse’ packages, v. 2.0.0^88^ and with statistics performed using the ‘rstatix’ package, v. 0.7.2^89^. All ecological indices were calculated using the vegan package v. 2.6-2^90^. Figures were produced with the ‘ggplot2’ package, v. 3.4.1^91^ with the exception of the maps that were produced using QGIS, v. 3.18.3^92^.

### Proteomic screening

Before peptide extraction all specimens were recorded using a modified faunal and taphonomic database to record a similar range of attributes as in the zooarchaeological analysis and following previous approaches^14,26,93,94^. A small bone splinter (~5 mg) was removed from each specimen, and subsequent ZooMS extraction was conducted at the palaeoproteomics lab at the Max Planck Institute for Evolutionary Anthropology in Leipzig (Germany). In total, 536 morphologically unidentified faunal remains were processed following existing protocols^95,96^. Empty wells were processed as laboratory blanks alongside the bone samples to assess potential contamination by non-endogenous peptides. All spectra were empty of collagenous peptides, excluding the possibility of laboratory or storage contamination.

All matrix-assisted laser desorption ionization (MALDI) spectra were automatically acquired at the Ecole Supérieure de Physique et Chimie industrielle (Paris, France) with an AB SCIEX 5800 MALDI-TOF spectrometer in positive reflector mode. Before sample acquisition, an external plate model calibration was achieved on 13 adjacent mass spectrometry (MS) standard spots with a standard peptide mix (Proteomix Peptide calibration mix4, LaserBioLabs). The calibration is validated according to the laboratory specifications (resolution above 10,000 for 573 Da, 12,000 for 1,046 Da and 15,000 to 25,000 for other masses, error tolerance <50 ppm). For MALDI MS sample measurements, laser intensity was set at 50% after optimization of signal-to-noise ratio on several spots, then operated at up to 3,000 shots accumulated per spot and covering a mass-to-charge range of 1,000 to 3,500 Da.

The triplicate data files obtained from the MALDI were merged in R using the packages MALDIquant and MALDIquantForeign to smooth the intensity of the peaks (applying a moving average function), remove the baseline (using the TopHat method) and align the spectra (SuperSmoother, signal-to-noise ratio of 3). The three replicates are then summed into a single spectrum, and the baseline is removed once more using the TopHat approach. The obtained.msd files were analysed in the open source MS tool mMass (http://www.mmass.org/). Glutamine deamidation values were calculated using the Betacalc3 package^97^.

SPIN is a shotgun proteomics workflow for analysing archaeological bone by liquid chromatography-tandem MS^98^. Here we applied SPIN to all the morphologically unidentifiable bone fragments recovered from the 2016–2022 excavations from layer 8 (*n* = 212) following existing methodologies^7,98^.

### sedaDNA

A total of 26 sediment samples were collected from layers 7 to 12 during excavations in 2020–2021 from the stratigraphic profile (see Supplementary Table 4 for samples per layer and year collected). Each sample was collected in a sterile manner, with the individuals collecting the samples wearing sterile gloves, a facemask, hairnet and clean room suit. A sterile scalpel was used to first remove a few millimetres of the exposed profile, and a second, fresh sterile scalpel was then used to collect at least 1 g of sediment in sterile 5 or 15 ml screw-cap tubes. The collected samples were then sealed in sterile plastic bags and transported back to a designated clean room at the Max Planck Institute for Evolutionary Anthropology for further processing.

In the clean room, sub-samples of ~50 mg were taken from each sample for automated DNA extraction (ref. ^99^; using buffer ‘D’) and single-stranded DNA library prep^100^. Negative controls were included for each of the extraction and library preparation steps. The resulting libraries were then enriched for a selection of 242 mammals^101^ via automated singleplex hybridization capture as described in ref. ^102^. Five microlitres of each enriched library were pooled in sets of 15 to 69 with libraries (including controls) from other projects for sequencing. Sequencing was performed on the Illumina MiSeq platform with Bustard used for basecalling.

The resulting sequencing data were processed following a previously published mitochondrial sediment DNA pipeline^103^. In brief, leeHom (v. 1.1.5)^104^ (https://bioinf.eva.mpg.de/) was used to merge overlapping paired-end sequences into single sequences that were then mapped to 242 mammalian mitochondrial genomes. Reads that were shorter than 35 bp, unmapped or could not be merged were then removed. In addition, sequences seen only once were removed, and a single sequence was retained from duplicate sequences. BLAST (v. 2.9.0)^105^ and MEGAN (v. 0.0.12)^106^ were then used to assign the remaining unique sequences to the family level. Within each family assignment, sequences were mapped to all available reference mitochondrial genomes per family. In this step PCR duplicates were removed using bam-rmdup (v. 0.2) (https://github.com/mpieva/biohazard-tools), and only sequences with a mapping quality of at least 25 were retained. The reference genome with the most aligned sequences was then used for generation of summary statistics and aDNA authentication (Supplementary Table 5). Taxa were identified as ancient if they met the following criteria: (a) at least 1% of total taxonomically identified sequences were assigned to the taxon in question, (b) have significantly higher than 10% C-to-T substitutions (based on 95% binomial confidence intervals) on one or both termini and (c) the fragments cover at least 105 base pairs of the reference mitochondrial genome.

### Stable isotope methodology

Approximately 400–600 mg material was sampled from each faunal specimen using a dentistry drill and diamond cutting disc, after surface removal via a sandblaster. Smaller samples of 55–160 mg were removed from the hominin bones. Collagen was extracted using the protocol described in refs. ^107,108^. Briefly, the sample chunks were demineralized in HCl 0.5 M at 4 °C until soft and CO_2_ effervescence had stopped, treated with NaOH 0.1 M for 30 min to remove humic acid contamination and then re-acidified in HCl 0.5 M. The samples were gelatinized in HCl pH3 (75 °C for 20 h for large samples and 70 °C for 2–6 h for small samples). The solubilized gelatin was then filtered to remove particles >60–90 µm (Ezee filters, Elkay Labs) and ultrafiltered to concentrate the >30 kDa fraction (Sartorius VivaSpin Turbo 15). Filters were pre-cleaned before use^109^. Finally, the >30 kDa fraction was lyophilized for 48 h, and the collagen was weighed to determine the collagen yield as a percentage of the dry sample weight.

Approximately 0.4–0.5 mg of collagen was weighed into tin capsules using an ultramicrobalance and measured on a Flash 2000 Organic Elemental Analyser coupled to a Delta XP isotope ratio mass spectrometer via a Conflo III interface (Thermo Fisher Scientific). Stable carbon isotope ratios were expressed using the delta notation (*δ*) relative to Vienna Peedee Belemnite (VPDB), and stable nitrogen isotope ratios were measured relative to AIR. The stable isotope delta values were two-point scale normalized using international reference materials IAEA-CH-6 (sucrose, *δ*^13^C = −10.449 ± 0.033‰), IAEA-CH-7 (polyethylene, δ^13^C = −32.151 ± 0.050‰), IAEA-N-1 (ammonium sulfate, *δ*^15^N = 0.4 ± 0.2‰) and IAEA-N-2 (ammonium sulfate, *δ*^15^N = 20.3 ± 0.2‰). Two in-house quality control standards were used to quality check the scale normalization and evaluate analytical precision: (1) EVA-0012 methionine (Elemental Microanalysis), *n* = 60, *δ*^13^C = −28.05 ± 0.06‰ (1 s.d.), *δ*^15^N = −6.41 ± 0.07‰ (1 s.d.); and (2) EVA MRG pig gelatin, *n* = 61, *δ*^13^C = −19.76 ± 0.25‰ (1 s.d.) and *δ*^15^N = 4.94 ± 0.12‰ (1 s.d.). This compares well to the long-term average values of *δ*^13^C = −28.0 ± 0.1‰ (1 s.d.) for EVA-0012 and *δ*^13^C = −19.7 ± 0.3‰ (1 s.d.) for EVA MRG, and *δ*^15^N = −6.4 ± 0.1‰ (1 s.d.) for EVA-0012 and *δ*^15^N = 5.0 ± 0.1‰ (1 s.d.) for EVA MRG.

The quality of the collagen extracts was assessed based on the yield, with minimum ~1% required. The elemental values (C%, N%, C:N) were compared to ranges of modern mammalian collagen (C, 30–50%; N, 10–17%), with C:N values of ~3.2 considered well preserved^110,111^ and with extracts falling outside the range of 2.9–3.6 excluded from analysis^112^. All extracts fell within accepted ranges and so were considered suitable for palaeodietary reconstruction (Extended Data Table 5).

### Reporting summary

Further information on research design is available in the Nature Portfolio Reporting Summary linked to this article.

## Supplementary information

Supplementary Information Suppementary Figs. 1–4 and accompanying discussion of additional isotope data.

Reporting Summary

Supplementary Data 1 All supplementary tables and data.

## Figures and Tables

**Extended Data Table 1:**
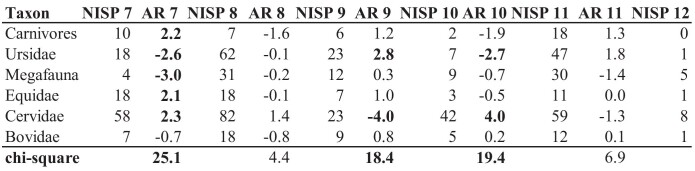
Chi-square test with adjusted residuals (AR) for Number of identified specimens (NISP) by layer and major taxon. AR refer to the layer to the left of the residuals column and should be read as standard normal deviates^117,118^

Significant values are >1.96 and are highlighted in bold. Chi square tests for taxon vs. Layer (Layer 12 vs Layer 11 χ2(5, n = 193) = 6.9, p = 0.23; Layer 11 vs Layer 10 χ2(5, n = 245) = 19.4, p = 0.001; Layer 10 vs. Layer 9 χ2(5, n = 148) = 18.4, p = 0.002; Layer 9 vs. Layer 8 χ2(5, n = 298) = 4.4, p = 0.5; Layer 8 vs. Layer 7 χ2(5, n = 333) = 25.1, p = 0.0001).

**Extended Data Table 2:**
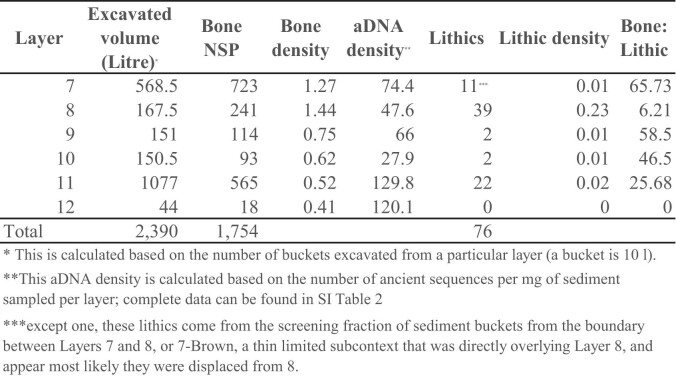
Bone, aDNA and lithic find densities from the 2016-2022 excavations at Ranis (Layers 12-7)

**Extended Data Table 3:**
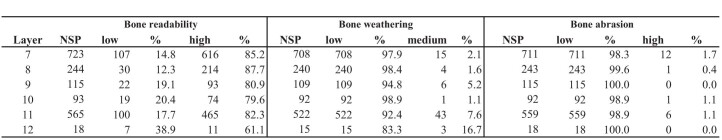
Macroscopic indicators of bone surface preservation

Bone readability is based on the proportion of original bone surface remaining and is based on Smith et al^14^ low includes 0% and <50%; high includes >50% and 100%; bone weathering is based on Behrensmeyer^71^; low weathering includes Stages 0 and 1, while medium weathering includes Stages 2 and 3; Bone abrasion is based on the proportion of the bone surface covered by the phenomenon and is based on Smith et al.^14^ low includes 0% and <50%; high includes >50% and 100%.

**Extended Data Table 4:**
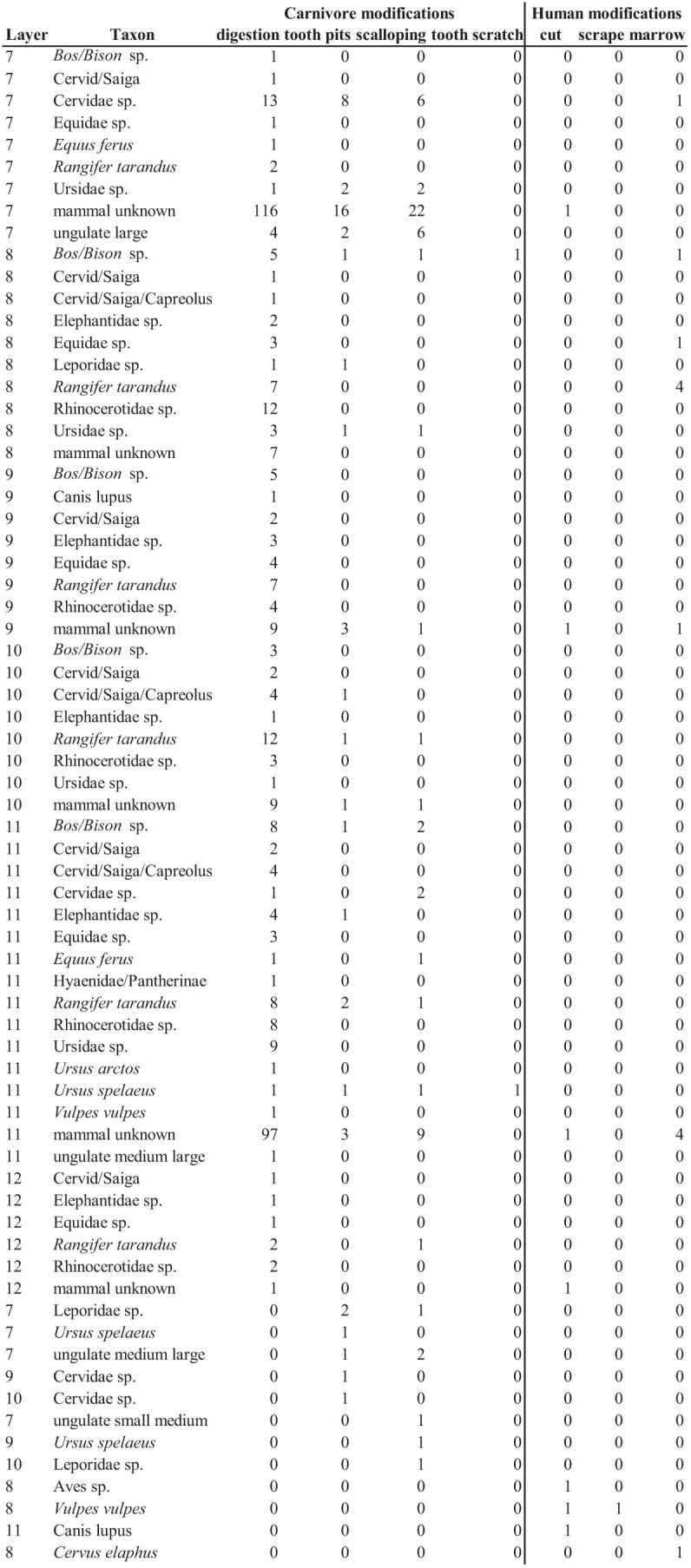
Bone surface modifications observed across the bone fragments recovered from Ilsenhöhle in Ranis

(broken down by species); these figures included modified specimens identified through both comparative morphology and ZooMS.

**Extended Data Table 5:**
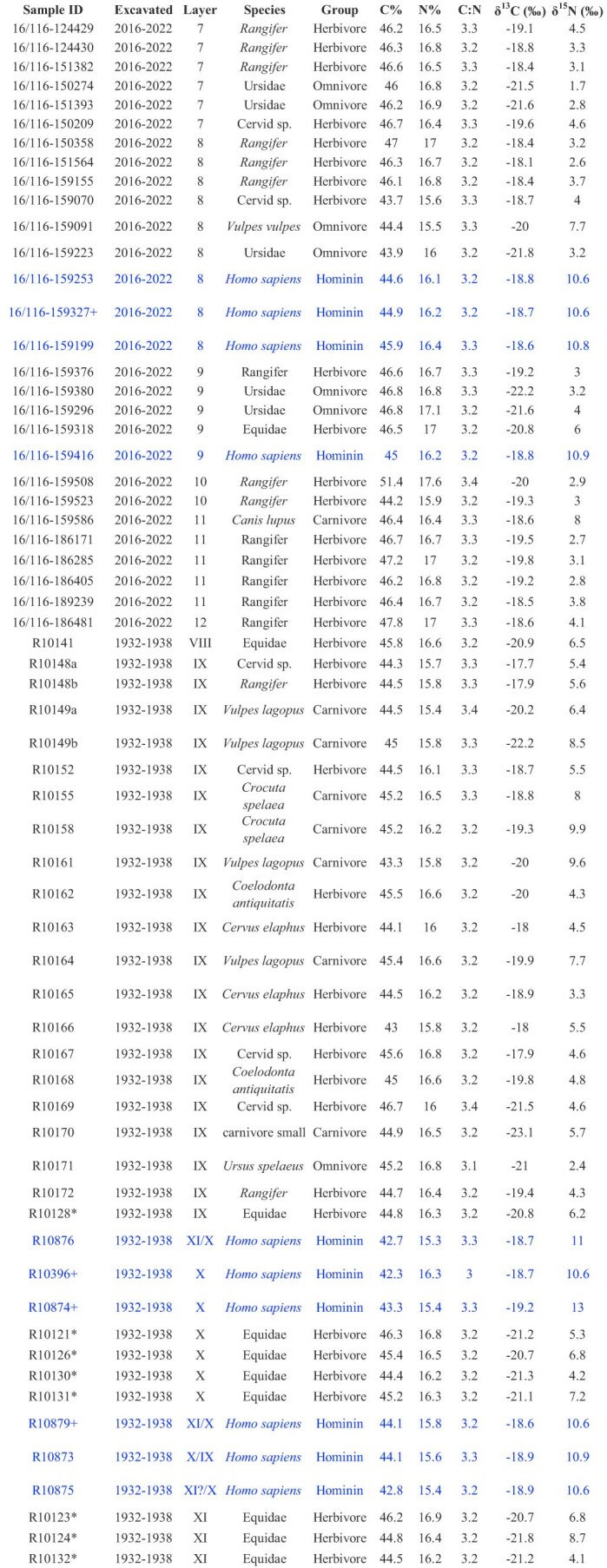
Bulk collagen C and N stable isotope data from Ranis

The cross (+) indicates the *Homo sapiens* bones where mtDNA indicates they could derive from the same individual or maternal relations. Equid samples marked with an asterisk (*) are reported in Pederzani et al.^13^, and have been directly dated to the same time period as Layers 11-7 from the 2016-2022 excavation.

**Extended Data Table 6:**
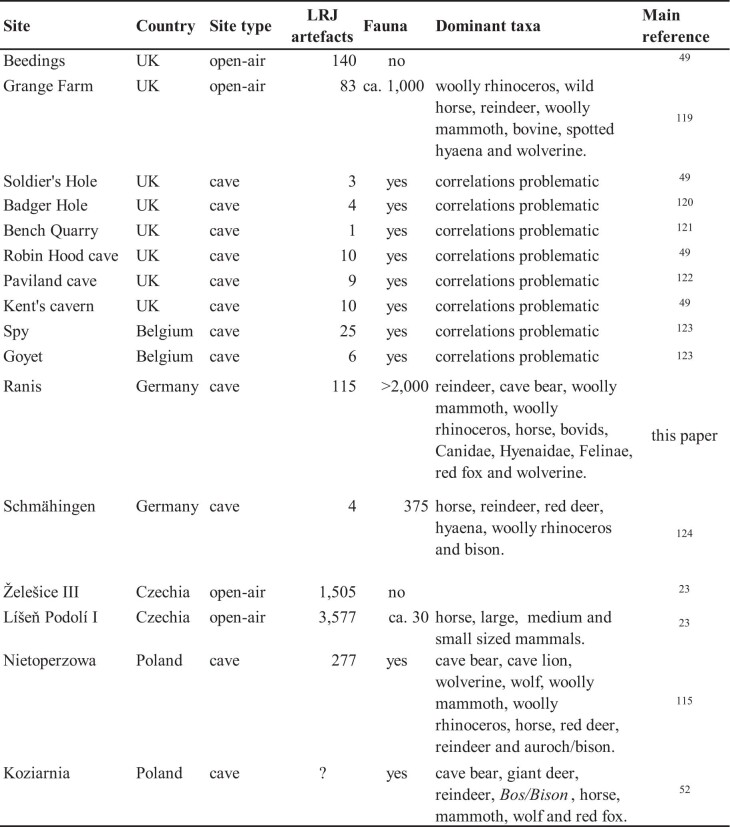
Overview of the main LRJ find spots and their associated faunal remains^23,49,52,115,119–124^

## Data Availability

The MS proteomics data have been deposited to the ProteomeXchange Consortium via the PRIDE^113^ partner repository under accession code PXD-043272. The MALDI-TOF.mzml and.msd type files included in this study are available at 10.5281/zenodo.8063812. The raw sequencing aDNA data of single-stranded libraries enriched for mammalian mtDNA from the 26 sediment samples are publicly available on the European Nucleotide Archive (PRJEB67902). Isotope data are available in Extended Data Table 5 and the Supplementary Information.

## Code availability

The R code associated with this work is publicly available through OSF at https://osf.io/aez4v/.
