# Supplementary material for: The ecology, subsistence and diet of ~45,000-year-old Homo sapiens at Ilsenhöhle in Ranis, Germany
Source: Nat Ecol Evol. 2024 Jan 31;8(3):564–77. doi: 10.1038/s41559-023-02303-6 (PMC10927544; doi:10.1038/s41559-023-02303-6)
Supplement: Supplementary file 1 — Suppementary Figs. 1–4 and accompanying discussion of additional isotope data. [file 41559_2023_2303_MOESM1_ESM.pdf]

nature ecology & evolution

Article https://doi.org/10.1038/s41559-023-02303-6

The ecology, subsistence and diet of ~45,000-year-old *Homo sapiens* at Ilsenhöhle in Ranis, Germany

In the format provided by the authors and unedited

Table of Contents

1. Supplementary Tables 2
2. Supplementary Figure 2
3. Bulk carbon and nitrogen analysis 3

1. Supplementary Tables

All Supplementary tables are contained in the associated excel workbook Smith_et_al_SI.

2. Supplementary Figure

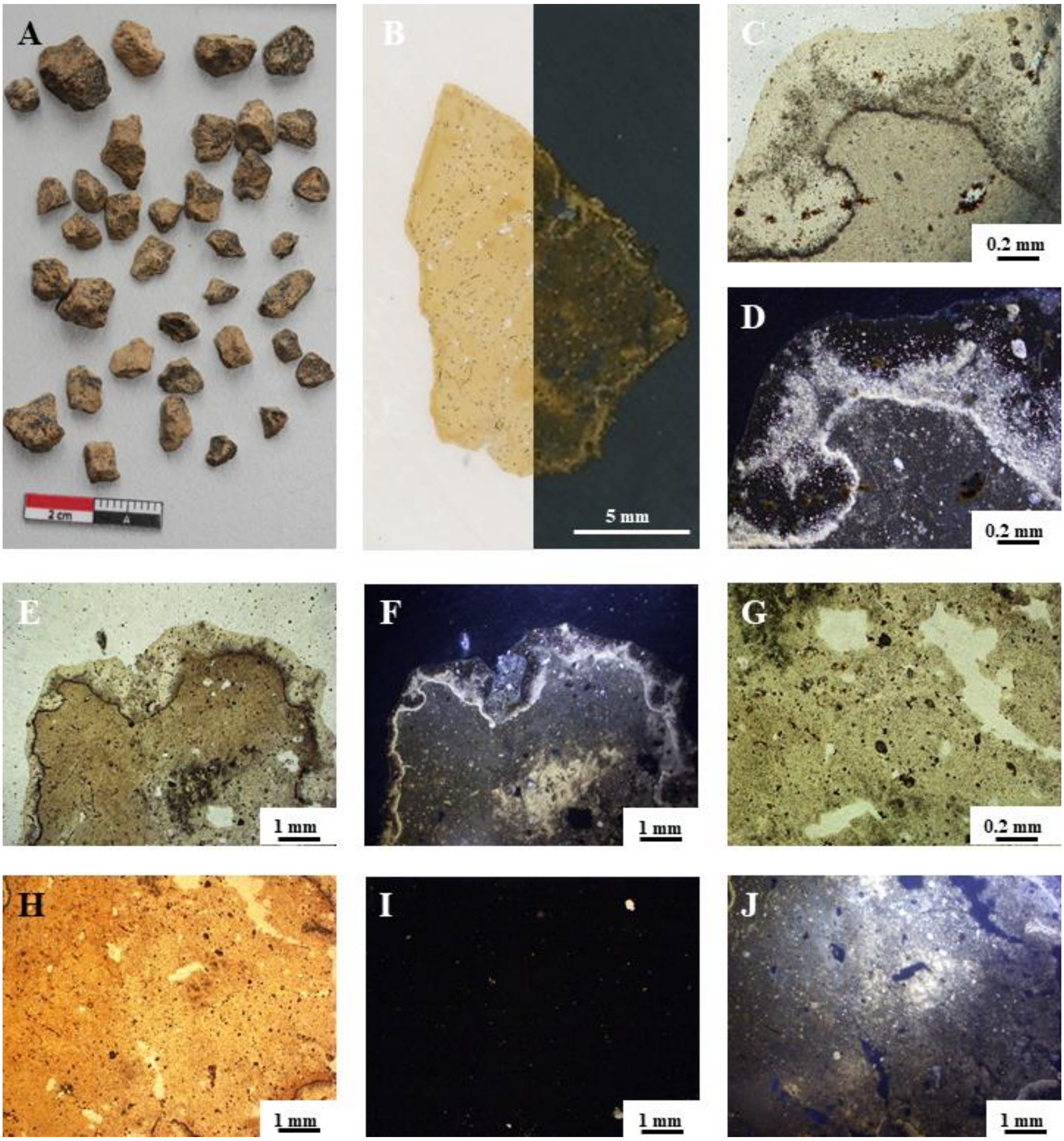

Supplementary Figure 1: Micromorphological analyses of a potential carnivore coprolite fragment from cave Ilsenhöhle at Ranis. Comparison with other micromorphological studies[1–3] supported this interpretation based on the characteristic presented in this figure. A. Coprolite fragments from layer 7. B: Scan of the coprolite fragment with closed (left) and open (right) lid showing a homogenous pale brown to yellow matrix and numerous voids. C to F: The edge of the

coprolite shows formation of a weathering rim in plane polarized light (PPL, C&E) and cross polarized light (XPL, D&F). Note also the inclusion of fine quartz grains G: Voids appear regularly and mainly present vughs. Typical gas vesicles or digested bone inclusions were not observed in this specimen. Microphoto in PPL. H to J: Groundmass in PPL (E), XPL (F) and oblique incident light (OIL, G) The groundmass is a speckled and isotropic.

3. Bulk carbon and nitrogen analysis

3.1 Stratigraphical context of the samples analysed for C and N isotopes at Ranis

From the 2016-2022 excavation, three *H. sapiens* were excavated from Layer 8 (16/116-159253; 16/116-159327; 16/116-159199), and one from Layer 9 (16/116-159416). Nine *Homo sapiens* bones from the 1932-1938 excavation were identified in boxes containing material from Layers IX/X (R10873), X (R10396; R10400; R10355; R10318; R10874), and X/XI (R10875; R10876; R10879), but all are associated with LRJ Layers 9 and 8 based on their direct ^{14}C dates[4]. mtDNA analysis indicates that five of the 13 *H. sapiens* bones may belong to the same or maternally related individuals (16/116-159327; R10396; R10355; R10874; R10879), although further nuclear DNA analysis is required to confirm this. Collagen was extracted from ten of the human bones for direct radiocarbon dating and stable isotope analysis, including four of the linked bones (16/116-159327, R10396, R10874, R10879), indicating that the human isotopic values presented could represent a minimum of seven individuals. Mylopotamitaki and colleagues[4] extracted collagen from 24 animal bones from Layers 12-7 from the 2016-2022 excavation for radiocarbon dating and Pederzani and colleagues[5] extracted collagen from 14 horse teeth from the 1932-1938 excavation and two horse bones from the 2016-2022 excavation, all of which were also directly radiocarbon dated. From the equid data published in Pederzani et al[5], we include here isotopic data from the specimens with ages overlapping with Layer 7 or older (n = 9). Carbon and nitrogen isotope ratios for these animals are listed in Extended Data Table 5 along with their associated layers. Additionally, we extracted collagen from various species found in Layer IX of the old excavation to provide a more detailed comparative dataset.

3.2 Diet of herbivorous species at Ranis

For Layers 9-7, we observe a separation in the feeding niches of equids compared to cervids, with equids showing higher $\delta^{15}N$ and lower $\delta^{13}C$ (Supplementary Figure 2, Supplementary Figure 3). Based on correlations with other environmental isotope systems, Pederzani et al. (see companion paper) suggested that this most likely reflects a specialized grazer feeding ecology of equids in a cold steppe environment. Reindeer, red deer and unidentified cervids show substantially higher $\delta^{13}C$ and lower $\delta^{15}N$ than equids, while woolly rhinoceros are intermediate between cervids and equids in both carbon and nitrogen stable isotope values (Supplementary Figure 2, Supplementary Figure 3). Higher $\delta^{13}C$ are expected for ruminant cervids, including reindeer, compared to non-ruminant equids and rhinoceros due to higher production of isotopically light methane in animals with ruminant digestive physiology[6]. High $\delta^{13}C$ values up to approximately -18 ‰ in reindeer are consistent with lichen consumption, which is common in reindeer, particularly in open environments and cold climatic episodes[7–10] that favor high lichen availability. While not as well-adapted to diets including larger amounts of lichen, other cervid species such as red deer have been documented to feed on lichen, especially during winter or in particularly cold years[11–13], explaining their isotopic niche overlap with reindeer in our data. Lichen consumption by cervids and an absence of any $\delta^{13}C$ below -22.5 ‰ support the prevalence of an open steppe/tundra environment with little woody cover during the LRJ at Ranis[14], matching well with a cold climatic episode reconstructed for this time.

3.3 Diets of omnivorous species at Ranis

Bear bones were identified in Layers 9-7 and the low $\delta^{15}N$ values of these samples are typical of cave bears[15]. In Layer 8, the red fox has a $\delta^{15}N$ value suggesting a carnivorous diet. However, the main prey of the red fox, mostly microfauna and birds, were not analyzed for this study, although some species have been identified throughout the lower horizons at Ranis (see Supplementary Table 2).

3.4 Diets of human and carnivorous species at Ranis

Compared to red fox (*Vulpes vulpes*), arctic foxes (*Vulpes lagopus*) show higher $\delta^{13}C$ and $\delta^{15}N$ values, which suggests a higher intake of meat, possibly from larger prey. Hyenas, wolves, and humans show similar $\delta^{13}C$ and $\delta^{15}N$ values, with the highest $\delta^{15}N$ values associated with humans, as typically observed in Pleistocene European sites[16–18]. Therefore, humans, hyenas, and wolves seem to hunt similar prey, including reindeer, rhinoceroses, and (to perhaps a lesser extent) horses.

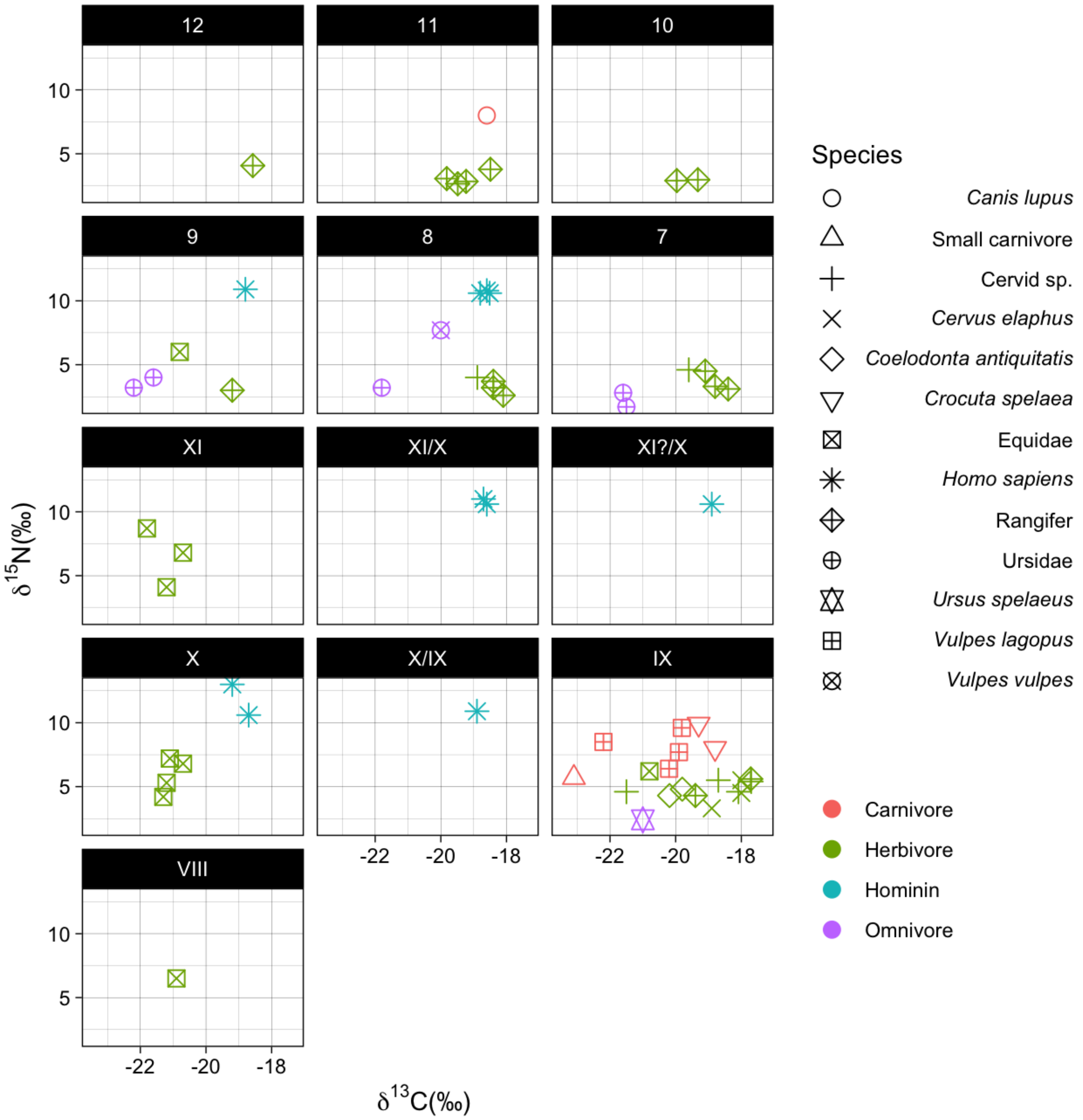

Supplementary Figure 2: Carbon and nitrogen isotope values of bulk collagen from Layers 12 - 7 from the 2016-2022 excavation and Layers XI - VIII from the 1932-1938 excavation at Ranis. Where more than one layer is indicated from the 1932-1938 collection (XI/X, XI?/X, X/IX), bones were stored in boxes containing material from more than one layer due to the excavation methods used at the time.

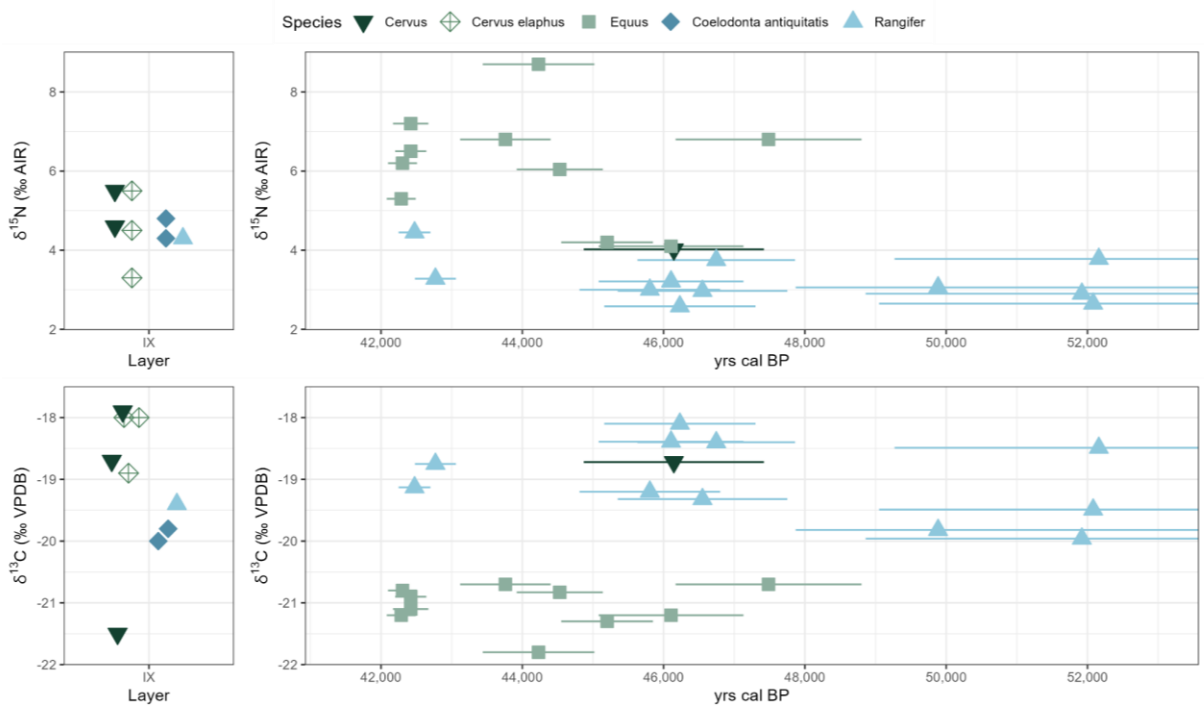

Supplementary Figure 3: Nitrogen isotope values of directly dated herbivore remains over time. Nitrogen isotope values of directly dated herbivore remains are remarkably high, and distinct from other species, indicating a specialized grazer feeding ecology. Carbon isotope separation with higher values in *Cervus* sp. and *Rangifer* show a stable grouping according to digestive physiology (ruminants vs non-ruminants). High $\delta^{13}C$ values of cervids suggest a contribution of lichen to the diet. Directly ^{14}C-dated data points (error bars show calibrated age range at 95% probability) from Mylopotamitaki et al. 2023 and Pederzani et al., 2023. Error ranges extending beyond the limit of the calibration curve are shown to extend to the limit of the plot without upper age limit. Total number of isotope samples (*n* = 33).

The nitrogen isotope ratios of human bulk collagen at Ranis are quite homogeneous, except for one bone sample with a higher $\delta^{15}N_{bulk}$ value of 13‰ (Figure S2, S3; $\delta^{15}N_{average}$ = 10.9 ± 0.73‰, n = 10, or $\delta^{15}N_{average}$ = 10.7 ± 0.17‰, n = 9, if individual R10874 is excluded). The elevated nitrogen isotope ratios observed in this individual may be attributed to their consumption of breast milk. Breast milk consumption is known to be associated with a ^{13}C enrichment ranging from 0 to 2‰. When considering the lower carbon isotope ratio in this individual compared to others in the *H. sapiens* population, several factors could contribute to this variance. It is plausible that the nursing parent relied less on reindeer meat as a dietary source, or the child may have experienced an extended period of catabolic stress[19,20]. Another possibility is that the child's diet consisted of ^{13}C-depleted and ^{15}N-enriched foods relative to those consumed by adults, such as horses, aquatic food or fermented meat (Figure 6)[21].

Compared to other hominins from Mousterian, Aurignacian, and Châtelperonnian periods, the Ranis *H. sapiens* have similar $\delta^{15}N$ values to Neanderthals, while their $\delta^{13}C$ values are similar to those of UP *H. sapiens* (Supplementary Figure 4A). We however need to look at local associated fauna to properly

interpret the diet of the humans, as environmental factors can strongly impact the isotope values of a food web[22]. UP *H. sapiens* generally exhibit much higher $\delta^{15}N$ values than local carnivores, while Neanderthals have more comparable values[16], which is interpreted as the signature of freshwater fish consumption by UP *H. sapiens*. This is not the case at Ranis, where the observed pattern is more similar to what is usually seen among European Neanderthals (Figure S4 and Supplementary Table 23[17]). The trophic level enrichment is particularly close to that of contemporaneous Neanderthals from Goyet, Belgium[23,24] (Supplementary Figure 4), but the local carnivores at Goyet showed lower $\delta^{15}N$ compared to those of Ranis (Supplementary Figure 4E). The trophic level enrichment is also similar to that observed for one individual of Buran Kaya (Supplementary Figure 4D) and Kostenki. Still, the trophic level enrichment is high, which could indicate the consumption of enriched ^{15}N foods such as horses, rhinos and potentially mammoths. We are also potentially missing foods eaten by these individuals at other seasonally occupied locations. *H. sapiens* usually exhibit higher $\delta^{13}C$ values than associated fauna, which could be the signature of the contribution of reindeer meat or aquatic foods to their diet. However, at Ranis, *H. sapiens* exhibit similar $\delta^{13}C$ values to the average herbivore values, suggesting the consumption of all mammal species. At Goyet, the main species contributing to the Neanderthals' diet were thought to be mammoths, rhinos, and reindeer. A more occasional mammoth consumption at Ranis could explain why carnivores and *H. sapiens* show closer $\delta^{15}N$ values compared to Goyet.

Based on the stable isotope analysis of *H. sapiens* bones at Ranis, it is likely that *H. sapiens* from Ranis consumed terrestrial meat from various mammal species, including rhinos, horses and reindeer, as part of their regular diet. The similarity in $\delta^{13}C$ values between the humans and herbivores suggests that the humans consumed a variety of mammal species rather than just one or two. Additionally, the trophic level enrichment of *H. sapiens* at Ranis but also at other locations (Supplementary Figure 4D) is similar to that of Neanderthals from other sites, suggesting that both groups were consuming similar prey. This interpretation is in contradiction with the hypothesis that European Palaeolithic *H. sapiens* were including substantial amounts of freshwater resources in their diet, but is in line with the latest CSIA-AA data supporting the hypothesis of UP *H. sapiens* diets relying on terrestrial resources[25].

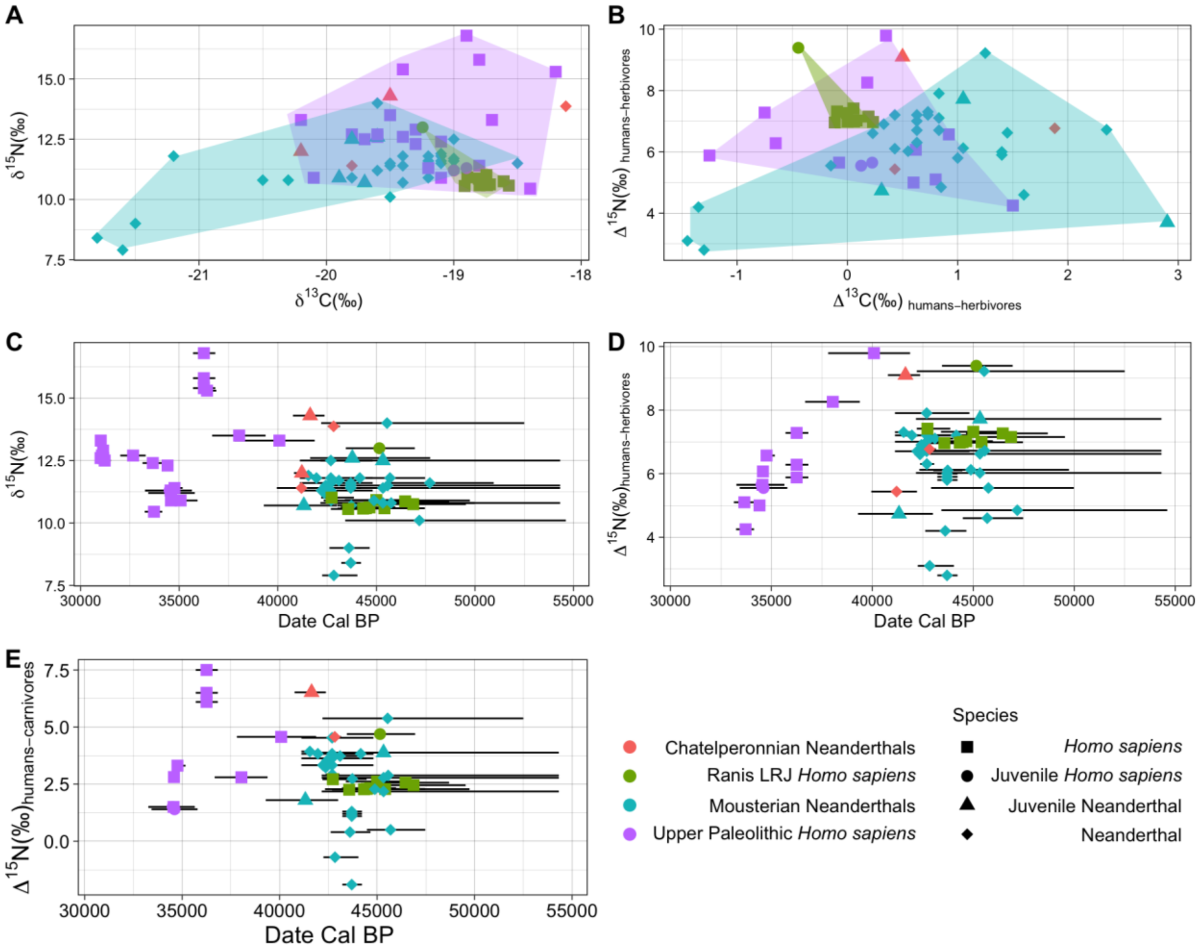

Supplementary Figure 4: Carbon and nitrogen isotope ratios of Neanderthals and *Homo sapiens* from European sites >31 ka cal BP. A. Raw carbon and nitrogen isotope ratios **B.** Offset between human individuals and associated herbivores for carbon and nitrogen isotope ratios **C.** Raw N isotope ratios over time in Aurignacian, Mousterian and Châtelperonnian sites in Europe **D.** Offset between human individuals and associated herbivores for nitrogen isotope ratios over time for the same time period. **E.** Offset between human individuals and associated carnivores for nitrogen isotope ratios over time in Upper Palaeolithic, Mousterian and Châtelperonnian sites in Europe. Number of samples (n = 66) and see Supplementary Table 23 and 24 for raw data and references. In 4C-4E, the error bars show the calibrated age range at 95% probability based on published data.

References

1. Brönnimann, D., Pümpin, C., Ismail-Meyer, K., Rentzel, P. & Égüez, N. Excrements of omnivores and carnivores. *Archaeological soil and sediment micromorphology*.
2. Horwitz, L. K. & Goldberg, P. A study of Pleistocene and Holocene hyaena coprolites. *J. Archaeol. Sci.*

3. Morley, M. W. *et al.* Hominin and animal activities in the microstratigraphic record from Denisova Cave (Altai Mountains, Russia). *Sci. Rep.* **9**, 13785 (2019).
4. Dorothea Mylopotamitaki, Marcel Weiss, Helen Fewlass, Elena Irene Zavala, Hélène Rougier, Arev Sümer, Mateja Hajdinjak, Geoff M. Smith, Karen Ruebens, Virginie Sinet-Mathiot, Sarah Pederzani, Florian Harking, Jesper V. Olsen, André Kirchner, Tobias Lauer, Mareike Stahlschmidt, Michael Hein, Sahra Talamo, Harald Meller, Holger Dietl, Jörg Orschiedt, Johannes Krause, Matthias Meyer, Frido Welker, Shannon P. McPherron, Tim Schüler, Jean-Jacques Hublin. Homo sapiens expanded into the northern European Plains by 45ka. *Nature*.
5. Sarah Pederzani, Kate Britton, Manuel Trost, Helen Fewlass, Nicolas Bourgon, Jeremy McCormack, Klervia Jaouen, Holger Dietl7 Hans-Jürgen Döhle, André Kirchner, Tobias Lauer, Shannon P. McPherron, Harald Meller, Dorothea Mylopotamitaki, Jörg Orschiedt7, Hélène Rougier, Karen Ruebens, Tim Schüler, Virginie Sinet-Mathiot1, Geoff M. Smith, Sahra Talamo, Thomas Tütken, Frido Welker, Elena I. Zavala Marcel Weiss, and Jean-Jacques Hublin. Early Homo sapiens dispersed into cold-arid steppes in central Europe. *Nature Ecology and Evolution*.
6. Cerling, T. E. & Harris, J. M. Carbon isotope fractionation between diet and bioapatite in ungulate mammals and implications for ecological and paleoecological studies. *Oecologia* **120**, 347–363 (1999).
7. Drucker, D. G., Hobson, K. A., Ouellet, J.-P. & Courtois, R. Influence of forage preferences and habitat use on 13C and 15N abundance in wild caribou (Rangifer tarandus caribou) and moose (Alces alces) from Canada. *Isotopes Environ. Health Stud.* **46**, 107–121 (2010).
8. Kristensen, D. K., Kristensen, E., Forchhammer, M. C., Michelsen, A. & Schmidt, N. M. Arctic herbivore diet can be inferred from stable carbon and nitrogen isotopes in C3 plants, faeces, and wool. *Can. J. Zool.* **89**, 892–899 (2011).
9. Drucker, D. G., Bridault, A. & Cupillard, C. Environmental context of the Magdalenian settlement in the Jura Mountains using stable isotope tracking (13C, 15N, 34S) of bone collagen from reindeer (Rangifer tarandus). *Quat. Int.* **272–273**, 322–332 (2012).
10. Rivals, F., Drucker, D. G., Weber, M.-J., Audouze, F. & Enloe, J. G. Dietary traits and habitats of the reindeer (Rangifer tarandus) during the Late Glacial of Northern Europe. *Archaeol.*

Anthropol. Sci. **12**, 98 (2020).

11. Gebert, C. & Verheyden-Tixier, H. Variations of diet composition of Red Deer (Cervus elaphus L.) in Europe. *Mamm. Rev.* **31**, 189–201 (2008).
12. Latham, J., Staines, B. W. & Gorman, M. L. Comparative feeding ecology of red (Cervus elaphus) and roe deer (Capreolus capreolus) in Scottish plantation forests. *J. Zool.* .
13. Ward, R. L. & Les Marcum, C. LICHEN LITTERFALL CONSUMPTION BY WINTERING DEER AND ELK IN WESTERN MONTANA. *Wildfire* **69**, 1081–1089 (2005).
14. Drucker, D. G., Bridault, A., Cupillard, C., Hujic, A. & Bocherens, H. Evolution of habitat and environment of red deer (Cervus elaphus) during the Late-glacial and early Holocene in eastern France (French Jura and the western Alps) using multi-isotope analysis (δ13C, δ15N, δ18O, δ34S) of archaeological remains. *Quat. Int.* **245**, 268–278 (2011).
15. Krajcarz, M. *et al.* Isotopic variability of cave bears (δ15N, δ13C) across Europe during MIS 3. *Quat. Sci. Rev.* **131**, 51–72 (2016).
16. Richards, M. P. & Trinkaus, E. Isotopic evidence for the diets of European Neanderthals and early modern humans. *Proceedings of the National Academy of Sciences* **106**, 16034–16039 (2009).
17. Bocherens, H. Neanderthal dietary habits: review of the isotopic evidence. *The evolution of hominin diets: integrating approaches to the study of Palaeolithic subsistence* (2009).
18. Jaouen, K. *et al.* Exceptionally high δ15N values in collagen single amino acids confirm Neandertals as high-trophic level carnivores. *Proc. Natl. Acad. Sci. U. S. A.* **116**, 4928–4933 (2019).
19. Neuberger, F. M., Jopp, E., Graw, M., Püschel, K. & Grupe, G. Signs of malnutrition and starvation--reconstruction of nutritional life histories by serial isotopic analyses of hair. *Forensic Sci. Int.* **226**, 22–32 (2013)
20. Mekota, A.-M., Grupe, G., Ufer, S. & Cuntz, U. Serial analysis of stable nitrogen and carbon isotopes in hair: monitoring starvation and recovery phases of patients suffering from anorexia nervosa. *Rapid Commun. Mass Spectrom.* **20**, 1604–1610 (2006)
21. Speth, J. D. Putrid meat and fish in the eurasian middle and upper paleolithic: Are we missing a

key part of neanderthal and modern human diet? *PaleoAnthropology*

22. Stevens, R. E. & Hedges, R. E. M. Carbon and nitrogen stable isotope analysis of northwest European horse bone and tooth collagen, 40,000BP–present: Palaeoclimatic interpretations. *Quat. Sci. Rev.* **23**, 977–991 (2004).
23. Wißing, C. *et al.* Stable isotopes reveal patterns of diet and mobility in the last Neandertals and first modern humans in Europe. *Sci. Rep.* **9**, 4433 (2019).
24. Wißing, C. *et al.* Isotopic evidence for dietary ecology of late Neandertals in North-Western Europe. *Quat. Int.* **411**, 327–345 (2016).
25. Drucker, D. G. *et al.* Isotopic analyses suggest mammoth and plant in the diet of the oldest anatomically modern humans from far southeast Europe. *Sci. Rep.* **7**, 6833 (2017).
