# Supplementary material for: The ecology, subsistence and diet of ~45,000-year-old Homo sapiens at Ilsenhöhle in Ranis, Germany
Source: Nat Ecol Evol. 2024 Jan 31;8(3):564–77. doi: 10.1038/s41559-023-02303-6 (PMC10927544; doi:10.1038/s41559-023-02303-6)
Supplement: Supplementary file 2 — Reporting Summary [file 41559_2023_2303_MOESM2_ESM.pdf]

nature portfolio

Corresponding author(s): Geoff M Smith
Last updated by author(s): Jul 6, 2023

Reporting Summary

Nature Portfolio wishes to improve the reproducibility of the work that we publish. This form provides structure for consistency and transparency in reporting. For further information on Nature Portfolio policies, see our Editorial Policies and the Editorial Policy Checklist.

Statistics

For all statistical analyses, confirm that the following items are present in the figure legend, table legend, main text, or Methods section.

n/a	Confirmed	
☐	☒	The exact sample size (n) for each experimental group/condition, given as a discrete number and unit of measurement
☐	☒	A statement on whether measurements were taken from distinct samples or whether the same sample was measured repeatedly
☐	☒	The statistical test(s) used AND whether they are one- or two-sided<br>*Only common tests should be described solely by name; describe more complex techniques in the Methods section.*
☒	☐	A description of all covariates tested
☒	☐	A description of any assumptions or corrections, such as tests of normality and adjustment for multiple comparisons
☐	☒	A full description of the statistical parameters including central tendency (e.g. means) or other basic estimates (e.g. regression coefficient) AND variation (e.g. standard deviation) or associated estimates of uncertainty (e.g. confidence intervals)
☒	☐	For null hypothesis testing, the test statistic (e.g. F, t, r) with confidence intervals, effect sizes, degrees of freedom and P value noted<br>*Give P values as exact values whenever suitable.*
☒	☐	For Bayesian analysis, information on the choice of priors and Markov chain Monte Carlo settings
☒	☐	For hierarchical and complex designs, identification of the appropriate level for tests and full reporting of outcomes
☒	☐	Estimates of effect sizes (e.g. Cohen's d, Pearson's r), indicating how they were calculated

Our web collection on statistics for biologists contains articles on many of the points above.

Software and code

Policy information about availability of computer code

Data collection	Data collected using freely available E4 software
Data analysis	Data analysed using R (4.2.0) and RStudio (2023.03.1)

For manuscripts utilizing custom algorithms or software that are central to the research but not yet described in published literature, software must be made available to editors and reviewers. We strongly encourage code deposition in a community repository (e.g. GitHub). See the Nature Portfolio guidelines for submitting code & software for further information.

Data

Policy information about availability of data

All manuscripts must include a data availability statement. This statement should provide the following information, where applicable:

- Accession codes, unique identifiers, or web links for publicly available datasets
- A description of any restrictions on data availability
- For clinical datasets or third party data, please ensure that the statement adheres to our policy

All data is available in manuscript and supplementary materials and code and data made available on publication.

Research involving human participants, their data, or biological material

Policy information about studies with human participants or human data. See also policy information about sex, gender (identity/presentation), and sexual orientation and race, ethnicity and racism.

Reporting on sex and gender	n/a
Reporting on race, ethnicity, or other socially relevant groupings	n/a
Population characteristics	n/a
Recruitment	n/a
Ethics oversight	n/a

Note that full information on the approval of the study protocol must also be provided in the manuscript.

Field-specific reporting

Please select the one below that is the best fit for your research. If you are not sure, read the appropriate sections before making your selection.

☐ Life sciences ☐ Behavioural & social sciences ☒ Ecological, evolutionary & environmental sciences

For a reference copy of the document with all sections, see nature.com/documents/nr-reporting-summary-flat.pdf

Ecological, evolutionary & environmental sciences study design

All studies must disclose on these points even when the disclosure is negative.

Study description	Analysis of bone remains through zooarchaeology and peptide mass fingerprinting (ZooMS, SPIN) combined with sedaDNA and bulk collagen stable isotopes to investigate the ecology, diet and subsistence of Homo sapiens at Ranis.
Research sample	Bone remains , sedaDNA and collagen bulk isotopes were analysed from all layers at Ranis but only Layers 7-12 are reported on here in line with sampling and reporting stategy of companion papers.
Sampling strategy	1754 bones from Layers 7-12 were studied using traditional morphological approaches to identify species and element and record all observable taphonomic attributes. Where it was not possible to identify these to species using morphology these specimens were analysed using zooarchaeology by mass spectrometry (ZooMS; n = 536) and on a more limited basis Species by Proteome INvestigation (SPIN, n = 212). sedaDNA samples were recovered from Layers 7-12 and analysed for the presence of animal and human DNA. Bulk collagen stable isotope values were obtained from radiocarbon samples (n = 54) taken from Layers 7-12 from both animal and human remains and combined with a selection of bulk collagen isotope samples from dated animal remains from old excavations at Ranis.
Data collection	G. Smith analysed the faunal material at the Max Planck Institute for Evolutionary Anthropology in Leipzig between 2017-2021. ZooMS analysis was undertaken at the Max Planck Institute for Evolutionary Anthropology in Leipzig between 2017-2021 by K. Ruebens, D. Mylopotamitaki, V. Sinet-Mathiot and F. Welker. DNA samples were collected between 2017-2021 by E. Zavala and analysed at the Max Plannck Institute for Evolutionary Anthropology in Leipzig. H. Fewlass sampled faunal remains from Ranis at the Max Plannck Institute for Evolutionary Anthropology in Leipzig between 2017-2021. M. Stahlschmidt analysed the coprolite material recovered between 2017-2021 at the Max Plannck Institute for Evolutionary Anthropology in Leipzig.
Timing and spatial scale	Bones and sediment samples were recovered from Ranis between 2015-2021 with material from Layers 7-12 recovered mainly between 2018-2021.
Data exclusions	No data excluded
Reproducibility	All data related to the analyses in these paper including the ZooMS spectra, DNA data and isotope values will be made available at publication.
Randomization	N/A
Blinding	N/A

Did the study involve field work? ☒ Yes ☐ No

Field work, collection and transport

Field conditions	Excavations at Ranis occurred during June/July between 2015-2021
Location	All material recovered from the cave Ilsenhöhle in Ranis, in Thuringia, Germany (50° 39′ 45,3″ N, 11° 33′ 53,5″ E)
Access & import/export	Material was studied and analysed at the Max Planck Institute for Evolutionary Anthropology in Leipzig and access arranged in collaboration between the Thüringer Landesamt für Denkmalpflege und Archäologie and Deptarment of Human Evolution.
Disturbance	The samples were obtained from excavations of the archaeological site. The area of the renewed excavations was kept as small as possible to reach the lowest layers following safety measures of stepped excavation levels.

Reporting for specific materials, systems and methods

We require information from authors about some types of materials, experimental systems and methods used in many studies. Here, indicate whether each material, system or method listed is relevant to your study. If you are not sure if a list item applies to your research, read the appropriate section before selecting a response.

Materials & experimental systems

n/a	Involved in the study	
☒	☐	Antibodies
☒	☐	Eukaryotic cell lines
☐	☒	Palaeontology and archaeology
☒	☐	Animals and other organisms
☒	☐	Clinical data
☒	☐	Dual use research of concern
☒	☐	Plants

Methods

n/a	Involved in the study	
☒	☐	ChIP-seq
☒	☐	Flow cytometry
☒	☐	MRI-based neuroimaging

Palaeontology and Archaeology

Specimen provenance	All bones were excavated from Ranis Cave, Germany, in a joint project of the Thüringer Landesamt für Denkmalpflege und Archäologie (Weimar, Germany) and the Department of Human Evolution at Max Planck Institute for Evolutionary Anthropology (MPI-EVA, Leipzig, Germany).
Specimen deposition	All specimens have been returned to the LDA and the TLDA, where they are curated under museum authority.
Dating methods	N/A

☒ Tick this box to confirm that the raw and calibrated dates are available in the paper or in Supplementary Information.

Ethics oversight	Permissions for destructive sampling were given by the LDA by the TLDA, who are the relevant archaeological authorities regulating protection of archaeological finds in Thuringia and Saxony-Anhalt, Germany.

Note that full information on the approval of the study protocol must also be provided in the manuscript.
